# 3D-QSAR-Based Pharmacophore Modeling, Virtual Screening, and Molecular Dynamics Simulations for the Identification of Spleen Tyrosine Kinase Inhibitors

**DOI:** 10.3389/fcimb.2022.909111

**Published:** 2022-06-30

**Authors:** Vikas Kumar, Shraddha Parate, Danishuddin, Amir Zeb, Pooja Singh, Gihwan Lee, Tae Sung Jung, Keun Woo Lee, Min Woo Ha

**Affiliations:** ^1^ Department of Bio & Medical Big Data (BK4 Program), Division of Life Sciences, Research Institute of Natural Science (RINS), Gyeongsang National University (GNU), Jinju, South Korea; ^2^ Division of Applied Life Science, Plant Molecular Biology and Biotechnology Research Center (PMBBRC), Gyeongsang National University (GNU), Jinju, South Korea; ^3^ The Interdisciplinary Graduate Program in Integrative Biotechnology and Translational Medicine, Yonsei University, Incheon, South Korea; ^4^ Laboratory of Aquatic Animal Diseases, Research Institute of Natural Science, College of Veterinary Medicine, Gyeongsang National University, Jinju-si, South Korea; ^5^ Jeju Research Institute of Pharmaceutical Sciences, College of Pharmacy, Jeju National University, Jeju, South Korea; ^6^ Interdisciplinary Graduate Program in Advanced Convergence Technology & Science, Jeju National University, Jeju, South Korea

**Keywords:** SYK inhibitor, 3D QSAR, pharmacophore, autoimmune diseases, molecular docking, MD simulation

## Abstract

Spleen tyrosine kinase (SYK) is an essential mediator of immune cell signaling and has been anticipated as a therapeutic target for autoimmune diseases, notably rheumatoid arthritis, allergic rhinitis, asthma, and cancers. Significant attempts have been undertaken in recent years to develop SYK inhibitors; however, limited success has been achieved due to poor pharmacokinetics and adverse effects of inhibitors. The primary goal of this research was to identify potential inhibitors having high affinity, selectivity based on key molecular interactions, and good drug-like properties than the available inhibitor, fostamatinib. In this study, a 3D-QSAR model was built for SYK based on known inhibitor IC_50_ values. The best pharmacophore model was then used as a 3D query to screen a drug-like database to retrieve hits with novel chemical scaffolds. The obtained compounds were subjected to binding affinity prediction using the molecular docking approach, and the results were subsequently validated using molecular dynamics (MD) simulations. The simulated compounds were ranked according to binding free energy (ΔG), and the binding affinity was compared with fostamatinib. The binding mode analysis of selected compounds revealed that the hit compounds form hydrogen bond interactions with hinge region residue Ala451, glycine-rich loop residue Lys375, Ser379, and DFG motif Asp512. Identified hits were also observed to form a desirable interaction with Pro455 and Asn457, the rare feature observed in SYK inhibitors. Therefore, we argue that identified hit compounds ZINC98363745, ZINC98365358, ZINC98364133, and ZINC08789982 may help in drug design against SYK.

## 1 Introduction

Kinases are a type of enzyme that can transfer high-energy phosphate from adenosine triphosphate to a substrate (Gagic et al; Duong-Ly and Peterson, 2013). The phosphorylation event modulates enzyme activity or communication with other molecules, leading to various biological reactions (Graves and Krebs, 1999). Tyrosine kinases are classified into receptor and non-receptor tyrosine kinases (NRTK) (Siveen et al., 2018). Spleen tyrosine kinase (SYK) is a cytoplasmic NRTK of hematopoietic lineage cells (Kobayashi et al., 1990). It was originally cloned from the cDNA of the porcine spleen, hence named SYK (Taniguchis et al., 1991). The SYK gene is situated at chromosome number nine, encoding a 629-amino-acid-long protein (Shao et al., 2021). The SYK gene is characterized by the C-terminal kinase domain ranging from 371 to 631 amino acids and two Src homology (SH2) domains: SH2 1 (15–107 residues) and SH2 2 (168–259 residues) at the N-terminal. The two SH2 domains are linked by interdomain A (108–167 residues), and the SH2 2 domain is linked to the kinase domain by interdomain B (260–370 residues) (Mócsai et al., 2010). SYK is present in an auto-inhibited form in the resting state; when antigen binds to the immunoreceptor, the Lyn protein tyrosine kinase gets activated and subsequently phosphorylates the immunoreceptor’s tyrosine-based activation motif (ITAM). As a result, SYK’s SH2 domains bind to phosphorylated ITAM, causing conformational changes in the kinase domain and eventually releasing the kinase (Kaur et al., 2013; Shao et al., 2021). The activated SYK then induces the signaling cascade to regulate the immune cell functions *via* phosphorylation of target cells. SYK is expressed in cell types such as B-cells, mast cells, macrophages, platelets, neutrophils, and cancer cells (Mócsai et al., 2010; Pamuk and Tsokos, 2010). SYK has been reported to contribute to various immune responses, counting cytokine production, histamine release, phagocytosis, reactive oxygen species (ROS), cell proliferation, differentiation, etc. (Currie et al., 2014; Thoma et al., 2015a). SYK acts as a pivotal protein in the downstream IgE/FcRI (Fc_ε_RI**)** signaling pathway to trigger allergic reactions (Nam et al., 2018; Hong et al., 2020). SYK is also important for the signaling cascade of B-cell receptors; a lack of SYK may result in the absence of mature B cells (Cheng et al., 1995; Turner et al., 1995). Recent studies indicated that SYK might regulate mitosis, differentiation, adhesion, and motility in non-hematopoietic cells such as epithelial and endothelial cells, and hepatocytes (Shao et al., 2021). Owing to the multiple roles of SYK in the cell, the dysfunction of SYK signaling has been reported in various diseases and disorders such as allergic rhinitis, asthma, autoimmune disease rheumatoid arthritis, psoriasis, systematic lupus erythematosus, leukemia, and lymphomas (Kaur et al., 2013). Therefore, several pharmaceutical companies, as well as academic institutions, have invested in the discovery of drug candidates against SYK. Several promising compounds based on naphthyridines, pyrimidine, imidazopyrimidine, and pyrido-thiazole scaffolds were identified during early drug development attempts against SYK but were not considered for clinical use due to poor pharmacokinetic profiles (Farmer et al., 2008; Hirabayashi et al., 2008; Liddle et al., 2011; Thoma et al., 2015b). However, some potential compounds such as PRT062607, R343, R112, and entospletinib (GS-9973) are among the SYK inhibitors that have entered clinical trials and are being examined for a variety of therapeutic uses (Liu and Mamorska-Dyga, 2017; Shao et al., 2021). Recently, the FDA has authorized the fostamatinib drug, developed by Rigel Pharmaceuticals to treat adult patients with immune thrombocytopenia (Mullard, 2018). A literature survey confirms that available drug options are limited against SYK as it has been reported in multiple diseases, and therefore there is a continuous need for SYK inhibitors. Over a thousand small compounds have been identified against SYK using various chemical bioassays. This information provides a good foundation for comprehending the structure–activity relationship between these molecules, making it simpler to discover novel SYK inhibitors. Therefore, the goal of the present study is to develop a pharmacophore model based on the quantitative structure–activity relationship (QSAR) using known inhibitors’ activity data. The pharmacophore models were validated *via* the test set and fisher randomization methods. The hypothesis was then used to screen the natural product database ZINC to retrieve potential compounds which could be used against SYK. We employed a molecular docking study to predict the binding affinity of the retrieved compounds with SYK, and results were subsequently authenticated by molecular dynamics simulations. Finally, hit compounds were selected on the basis of better binding free energy and key molecular interactions than reference drug fostamatinib and proposed as novel SYK inhibitors for further studies.

## 2 Materials and Methods

### 2.1 Collection of Dataset

The selection of chemical scaffolds is a decisive step for developing a 3D-QSAR pharmacophore model because this determines the characteristic of the pharmacophores generated (John et al., 2011). One of the essential requirements for generating 3D QSAR is experimentally tested compounds using the same bioassay (Sakkiah and Lee, 2012). We performed the literature search and selected 180 SYK inhibitors whose activity was determined using the same bioassay (Lucas et al., 2012; Padilla et al., 2013; Lucas et al., 2014). The 2D chemical structures of the inhibitors were dawn with BIOVIA draw v2021 and after that changed to 3D in Discovery Studio (DS) v19 (www.accelrys.com). Duplicate compounds were removed to ensure further statistical relevance, and compounds with diverse chemical scaffolds were selected. The three-dimensional conformation of inhibitors was energy minimized in DS with the *Steepest Descent* algorithm. The remaining compounds were divided into training and test sets following four orders of magnitude in their biological activities. The training set compounds were used to build the pharmacophore hypothesis, and the test set was used to validate the hypothesis (Kumar et al., 2015). The IC_50_ values of the selected training set compounds reported against SYK were in the range of 1 to 31,623 nmol/L. The training set compounds were classified into three categories as active (IC_50_ <10 nmol/L +++), moderately active (IC_50_ <100 nmol/L ++), and inactive (IC_50_ <1,000 nmol/L +), based on their IC_50_ values. Similar criteria were used for test set classification.

### 2.2 Pharmacophore Model Generation

Pharmacophore modeling is an effective and time-saving approach for identification of new scaffolds, and it may be constructed using either ligands or structure of the target biomolecule. The ligand-based hypothesis can be further developed using a common feature of most active ligands or using the structure–activity relationship of the training set compounds (Sakkiah et al., 2010; Kaserer et al., 2015; Mali and Pandey, 2021). In the current study, we utilized knowledge of available SYK inhibitors’ biological activity data to build a 3D QSAR pharmacophore model. The *3D QSAR Pharmacophore Generation* module of DS was used to create quantitative pharmacophores. To find the important characteristics in the training set compounds, the *Feature Mapping* module in DS was exploited. The knowledge of feature mapping results was subsequently utilized for the generation of the hypothesis. Before hypothesis generation, the uncertainty value was fixed at 3, representing the ratio of the real activities to the observed biological activity for each compound (Kumar et al., 2015). At the same time, other parameters were kept as default. The minimum and maximum numbers of features were set to 0 to 5 during the quantitative hypothesis generation. The best hypothesis was chosen among 10 generated models using the following criteria: highest correlation coefficient (R^2^), lowest total cost, fit values, and root mean square deviation (RMSD) (Sakkiah and Lee, 2012).

### 2.3 Hypothesis Validation

The selected hypothesis was validated using test set analysis and Fischer’s randomization test (Kumar et al., 2015). The statistical relevance of the hypothesis was assessed using Fischer’s method. The hypothesis is considered significant if the overall total cost is lower than the randomly generated hypothesis (Sakkiah and Lee, 2012; Kumar et al., 2015). Using confidence level 95%, the *Hypogen* algorithm of DS generates 19 random spreadsheets during the Fischer’s test. The test set approach was also employed to see if the selected hypothesis could correctly predict and categorize the chemical compounds based on their biological activity scale. Randomly 19 compounds were selected with four orders of magnitude from similar literature sources, used to generate the hypothesis. The test set compounds were then mapped on the selected hypothesis using the *Ligand Pharmacophore Mapping* function of DS with the *BEST* algorithm and flexible-fitting method (Kumar et al., 2015).

### 2.4 Virtual Screening of Natural Products in the ZINC Database

Virtual screening is a computational procedure commonly used to screen chemical compound libraries to filter false binders. We used our validated QSAR model to screen the natural products (144,766) in the ZINC database (https://zinc.docking.org/). Initially, a drug-like database was prepared using commonly used filters such as Lipinski’s Rule of Five (Ro5), Veber’s, and ADMET (absorption, distribution, metabolism, excretion, and toxicity) to reduce computational cost (Lipinski et al., 2001; Veber et al., 2002; Parate et al., 2021a). As explained in the previous literature, the aforementioned drug-like rules collectively oversee compounds’ proficient retrieval based on their pharmacokinetics and physiochemical properties. The drug-like database so obtained was screened to examine and retain only the natural products able to map chemical features of the QSAR model (Sundarapandian et al., 2010). Subsequently, the mapped ZINC natural compounds have proceeded to molecular docking with the 3D X-ray structure of the SYK protein.

### 2.5 Molecular Docking of Compounds With the SYK Domain

According to the extensive examination of published structures for SYK, there are 62 distinct inhibitor-bound structures deposited in the protein data bank (PDB). As a result, choosing an appropriate structure for molecular docking is a difficult choice; however, based on previous docking studies, researchers suggested a few key points to make a good selection, such as resolution of structure <2Å, binding with a well-known inhibitor, key molecular interaction, fewer missing residues, and mutations (Bender et al., 2021). Careful inspection of these parameters reveals that the recently published SYK structure bound with clinical candidate drug lanraplenib (PDB ID: 6VOV) is of a good choice for molecular docking-based study (Blomgren et al., 2020). The selected structure was obtained from PDB and prepared in DS using the *Clean Protein* module. The unwanted molecules were deleted, and gaps were filled. Subsequently, the structure was minimized using the *Steepest Descent* algorithm of DS and saved in PDB format for molecular docking (Kumar et al., 2021a; Mali and Pandey, 2022). The small molecules obtained from pharmacophore screening and REF drugs fostamatinib and lanraplenib were also minimized in DS using the *Steepest Descent* algorithm and saved in MOL2 format. In the current study, Genetic Optimization for Ligand Docking (GOLD v5.2.2) software was utilized to establish the molecular interactions between the potential inhibitors and the target receptor (Jones et al., 1997). First, the robustness of GOLD software was estimated before carrying out the docking experiment with crystallized ligand, lanraplenib, and the root mean square deviation was computed. The docking site was assigned as a 10-Å sphere by DS’s *Define and Edit Binding Site* module with X, Y, and Z coordinates of -13.06, 19.90, and -4.82, respectively. Following software robustness, the drug-like natural molecules were employed for docking utilizing the exact coordinates used for docking of lanraplenib. The first stage included docking-based virtual screening, where only one conformer per ligand was generated. The compounds exhibiting better docking results than lanraplenib and fostamatinib (REF inhibitors) were retained. The second stage comprised exhaustive docking of the retained compounds by generating 10 conformers per ligand. The compounds indicating higher GoldScores, lower ChemScores than REF inhibitors, and promising interactions with the active site residues within the SYK domain were further studied under atomic-level molecular simulations (Kumar et al., 2021b; Parate et al., 2021b).

### 2.6 Molecular Dynamics Simulations

The stability and binding flexibility of the selected protein-ligand docking complexes were studied under real time using molecular dynamics simulations. To accomplish this task, Groningen Machine for Chemical Simulations (GROMACS v5.1.5) was used (Van Der Spoel et al., 2005; Pronk et al., 2013). The simulation parameter files for SYK and potential inhibitor compounds were generated by Chemistry at Harvard Macromolecular Mechanics forcefield (CHARMm27) and SwissParam, respectively (Sapay and Tieleman, 2011; Zoete et al., 2011). The TIP3P system was employed for hydration of the protein–ligand complex throughout the simulation run. Further, the counter ions were used to neutralize the simulation box. Consequently, energy minimization equilibration using NVT and NPT was performed. The molecular simulations were conducted under periodic boundary conditions to reduce the edge effects (Kumar et al., 2021c;  Kumar et al., 2021b). The MD simulation trajectories were assessed with the DS and VMD program (Humphrey et al., 1996).

### 2.7 Binding Free Energy Calculations

The MD simulation results were subsequently subjected to predict the binding free energy (ΔG) using a computationally well-founded method, molecular mechanics Poisson–Boltzmann surface area (MM-PBSA) (Kollman et al., 2000). The *g_mmpbsa* plugin package in GROMACS was utilized to predict the ΔG of the protein–ligand complex in the solvent, which can be defined as (Kumari et al., 2014):


$$\Delta G_{bind}=G_{complex}-\left(G_{protein}+G_{ligand}\right)$$

where G_complex_ is the protein–ligand complex’s total free energy, whereas G_protein_ and G_ligand_ are the over-all free energies of the protein and ligand alone in solvent.

## 3 Results and Discussion

### 3.1 Construction of the Pharmacophore Model

**Figure 1:**
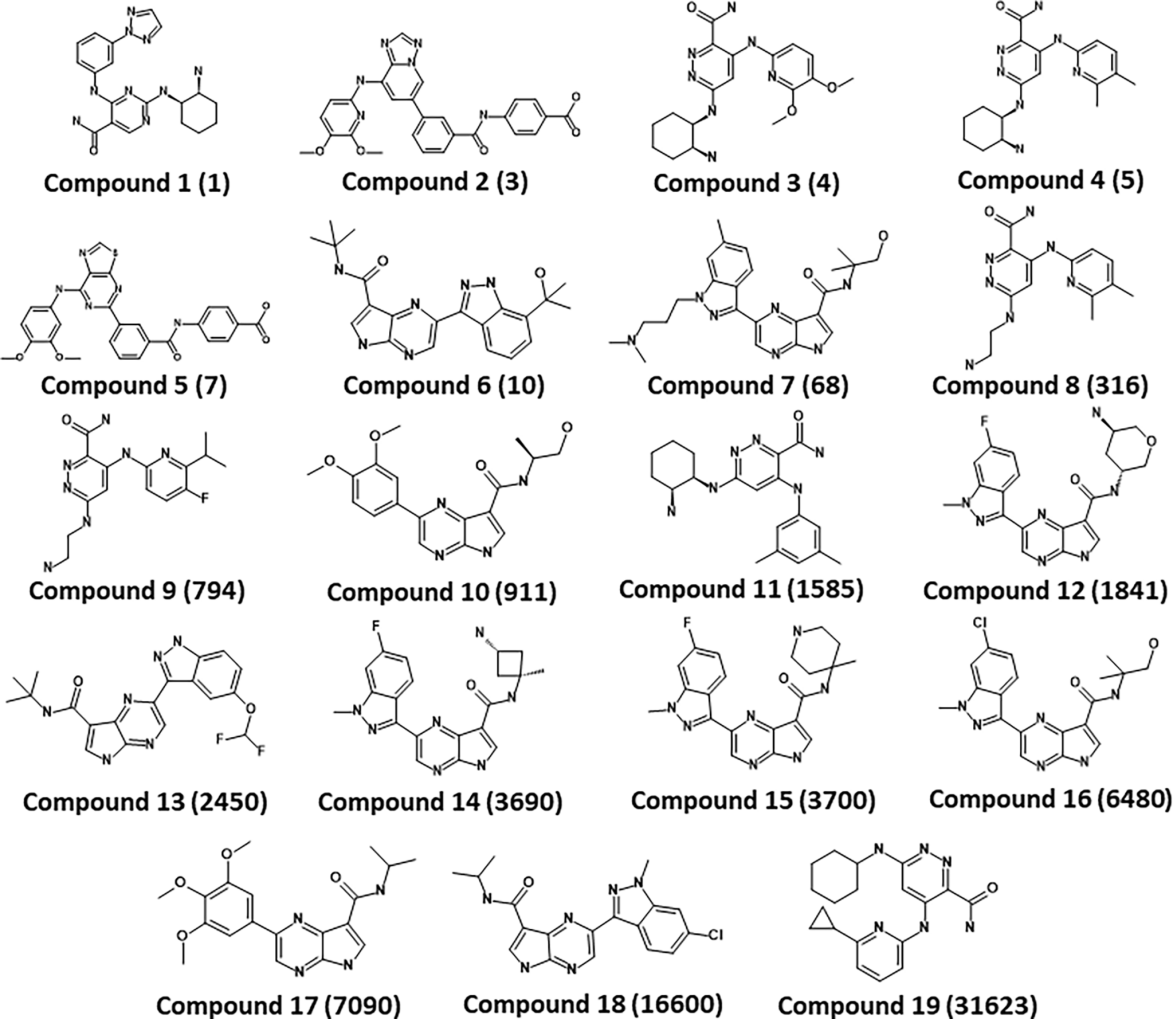
Chemical structures of the SYK inhibitors in the training set. The inhibitory activity of the compounds is shown in parenthesis (IC_50_ nmol/L).

**Figure 2:**
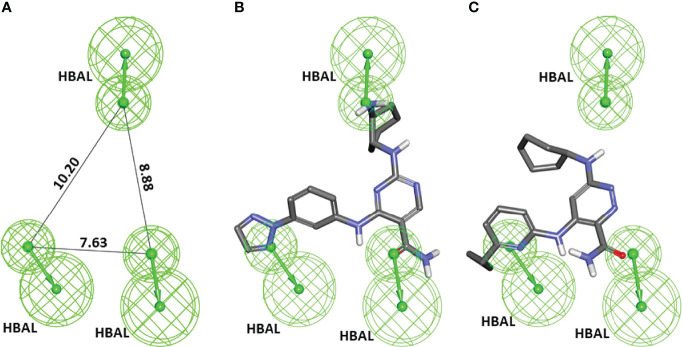
**(A)** The best pharmacophore model, Hypo1, shows chemical features and distance constraints Å. Hypo1 contains three hydrogen bond acceptor lipid (HBAL) features. **(B, C)** The pharmacophore mapping results of Hypo1 on the training set’s most active and least active compound, respectively.

**Figure 3:**
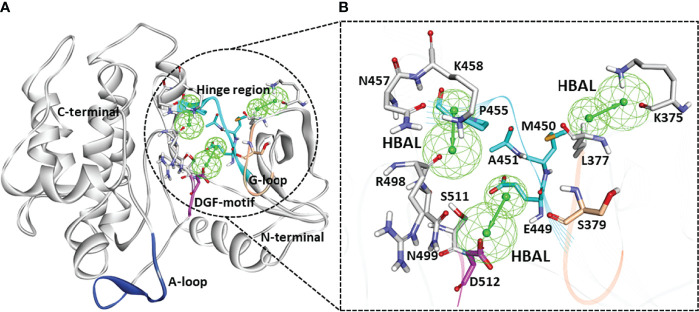
**(A)** The ribbon diagram of the SYK protein showing an overlay of Hypo1 inside the active site of different SYK domains. The regions of the SYK active site such as the hinge region (448–455), glycine-rich loop (378–383), DFG motif (512–514), and the activation loop (520–534) were highlighted. **(B)** The mapping of Hypo1 features with SYK active site residues. The SYK residues were shown with a stick representation in different colors: hinge (cyan), G-loop (teak), DFG motif (pink), and other active site surrounding resides (gray).

A series of SYK inhibitors tested through the same biological assay method were collected from different literature sources (Lucas et al., 2012; Padilla et al., 2013; Lucas et al., 2014). A training set of 19 compounds with diverse scaffolds was carefully compiled to construct the pharmacophore model. The 2D structures and the inhibitory concentrations of the training set compounds against SYK are shown in **Figure 1**. Based on the experimental inhibitory concentration of compounds, the training set was classified as active (IC_50_ <10 nmol/L +++), moderately active (IC_50_ <100 nmol/L ++), and inactive (IC_50_ <1,000 nmol/L +). Before the generation of the hypothesis, the *Feature Mapping* module of the DS was exploited using training set compounds as an input to get an idea about the proportion of chemical features present in compounds. Rationally, the five most common features, viz., hydrogen bond donor (HBD), hydrogen bond acceptor (HBA), hydrogen bond acceptor lipid (HBAL), ring aromatic (RA), and hydrophobic (HYP), were carefully chosen from feature mapping results for the hypothesis generation. Subsequently, the *DS’s 3D QSAR Pharmacophore Generation module* was utilized to generate the set of pharmacophore models. The program generates 10 hypotheses, each with its own set of statistical parameters, including total cost, cost difference, RMSD, correlation, Max Fit, and features (**Table 1**). According to the previous studies, the best hypothesis should have fit value >9, maximum cost difference, least total cost values, high correlation coefficient, and lowest root mean square deviation (RMSD) values (Debnath, 2002; John et al., 2011; Kumar et al., 2015). It is shown in **Table 1** that except for Hypo9, and Hypo10, all others display a fit value greater than nine. Furthermore, Hypo8 showed the highest fit value of 11.3, followed by Hypo5, Hypo3, Hypo2, and Hypo1. **Table 1** also shows that the hydrogen bond acceptor lipid (HBAL) feature was dominantly found in all generated hypotheses, suggesting that it is necessary for SYK inhibition. The detailed analysis of statistical parameters revealed that Hypo1 had a low total cost of 95.78, a maximum cost difference of 50.78, the least RMSD value (0.69), and a maximum correlation (0.98). Thus, Hypo1, which contains three features of HBAL, was selected as the best hypothesis (**Figure 2A**). To illustrate the predictive accuracy of Hypo1, regression analysis was employed. Hypo1 estimates the inhibitory value of the compounds in their activity range with minor deviations. One active and one moderately active compound was anticipated to be moderately active and inactive, respectively, as shown in **Table 2**. Furthermore, the training set’s most active compound (IC_50_ = 1 nmol/l) was found to be well-matched with the three chemical features of Hypo 1, although one HBAL feature was not well linked with the training set’s least active compound (IC_50_ = 32,000 nmol/L) (**Figures 2B, C**). The mapping difference between most active and least active compounds reveals the possible difference between their inhibitory activities against SYK. Additionally, we studied the possible mapping of the Hyop1 with SYK active site. **Figure 3A** displays the overlay of Hypo1 inside the active site of the SYK protein. The enlarged view in **Figure 3A** indicates that three HBAL features of Hypo1 may map with K375, R499, and DFG motif residue D512. In addition, the surrounding active site residues were also shown, which may participate in various hydrophobic interactions during protein-ligand binding (**Figure 3B**). The feature mapping results reveal that the Hypo1-filtered compound may target mentioned active site residues. The potential of Hypo1 to predict the activity similar to the experimental activity range and mapping with active site key residues indicates that Hypo1 may act as a good 3D query to identify novel compounds from drug-like databases.

**Table 1:** The statistical details of 10 Hypogen algorithm-generated pharmacophore hypotheses.

Hypo no.	Total cost	Δcost*^a^*	RMS*^b^*	Correlation (R^2^)	Max fit	Features*^c^*
Hypo1	95.78	50.78	0.69	0.98	9.35	HBAL, HBAL, HBAL
Hypo2	101.21	45.36	0.92	0.96	9.79	HBAL, HBAL, HBAL
Hypo3	105.72	40.85	1.42	0.87	10.39	HBAL, HBAL, HBD, HYP
Hypo4	106.64	39.93	1.32	0.90	9.03	HBA, HBA, HBAL
Hypo5	107.38	39.18	1.50	0.86	10.10	HBA, HBAL, HBD, HYP
Hypo6	107.46	39.10	1.27	0.91	9.52	HBAL, HBAL, HBD
Hypo7	108.12	33.44	1.36	0.89	9.17	HBA, HBA, HBAL
Hypo8	108.59	37.97	1.46	0.87	11.3	HBA, HBAL, HBD, HYP
Hypo9	108.75	37.81	1.41	0.88	8.95	HBAL, HBAL, HBAL
Hypo10	108.83	37.73	1.41	0.88	8.95	HBA, HBAL, HBAL

^a^Δcost is the difference between the null cost (146.57) and the total cost. ^b^RMS is root mean square deviation. ^c^HBAL, hydrogen bond acceptor lipid; HBD, hydrogen bond donor; HBA, hydrogen bond acceptor; HYP, hydrophobic.

**Table 2:** Detailed overview of actual biological activities of training set compounds and Hypo1-predicted activities.

Compound no.	Fit value	Experimental IC_50_ nmol/L	Predicted IC_50_ nmol/L	Error^a^	Experimental scale	Predicted scale^b^
1	9.10	1	2.5	+2.5	+++	+++
2	8.90	3	4.1	+1.4	+++	+++
3	8.44	4	12	+2.9	+++	+++
4	8.21	5	20	+4	+++	+++
5	8.80	7	5.1	-1.4	+++	+++
6	7.84	10	46	+4.6	++	++
7	7.80	68	51	-1.3	++	++
8	7.16	320	220	-1.4	++	++
9	6.38	790	1,300	+1.7	++	+
10	6.71	910	630	-1.4	++	++
11	6.22	1,600	1,900	+1.2	+	+
12	6.45	1,800	1,100	-1.6	+	+
13	6.12	2,400	2,500	+1	+	+
14	6.20	3,700	2,000	-1.8	+	+
15	6.17	3,700	2,200	-1.7	+	+
16	6.13	6,500	2,400	-2.8	+	+
17	6.21	7,100	2,000	-3.6	+	+
18	5.29	17,000	16,000	-1	+	+
19	5.30	32,000	16,000	-2	+	+

^a^Error, ratio of the (predicted IC_50_ to the experimental IC_50_ or its negative inverse if the ratio is <1). ^b^Activity scale: IC_50_ <10 nmol/L = +++ (active), 10 nmol/L ≤ IC_50_ < 1,000 nmol/L = ++ (moderate active), IC_50_ ≥ 1,000 = + (inactive).

### 3.2 Hypothesis Validation

**Figure 4:**
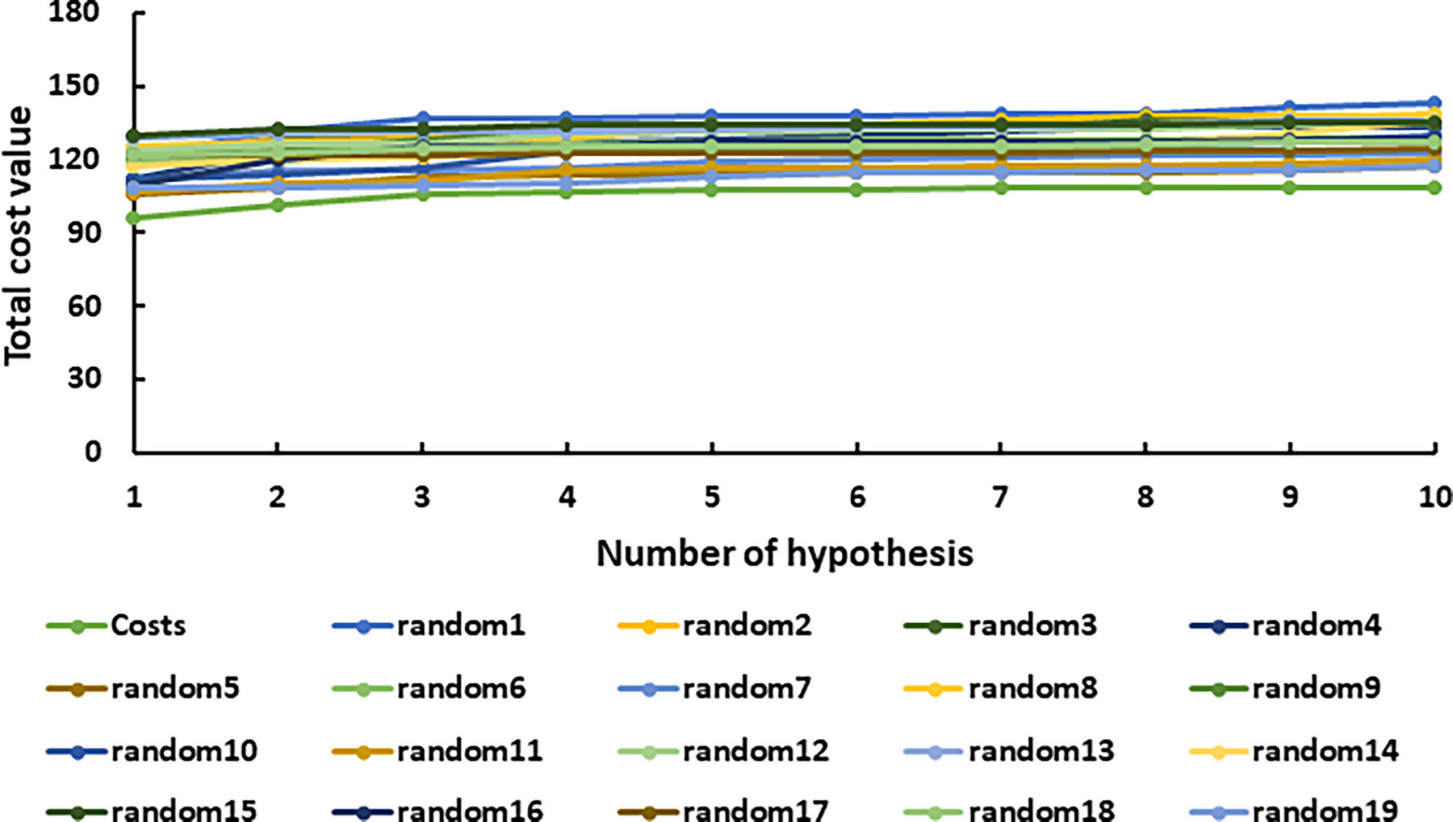
The graphical depiction of the total cost analysis of the initial spreadsheet (costs) and 19 random spreadsheets during Fischer’s randomization run. A confidence threshold of 95% was applied.

**Figure 5:**
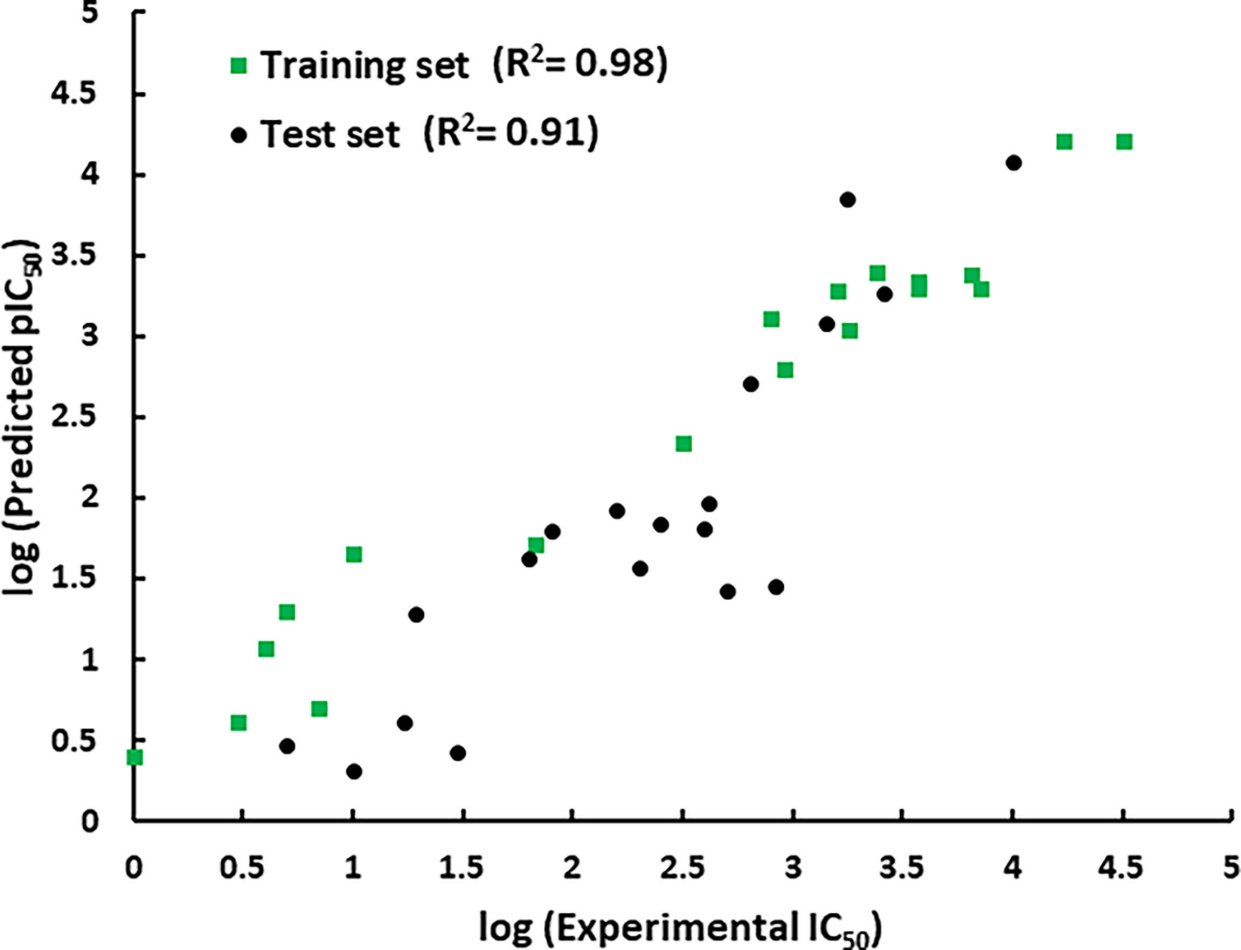
The graphical representation of the correlation between the training set and test set compounds’ experimental and Hypo1-predicted activities.

The representative hypothesis was evaluated *via* Fischer’s randomization test and test set validation (Kumar et al., 2015). In Fischer’s validation method, the statistical significance of the hypothesis was investigated by generating 19 random spreadsheets of the training set compounds using the actual biological activity values at the confidence level of 95%. It can be inferred from **Figure 4** that Hypo1 was not developed by chance, as Hypo1 showed the lowermost cost value among 19 randomly generated hypotheses. Additionally, the correlation of the randomly generated hypothesis was also compared with Hypo1, and it was observed that Hypo1 achieved the highest correlation among randomly generated hypotheses (**Figure S1**). The test set validation was performed using 19 structurally different compounds (**Table S1**). The inhibitory activity of the compounds in the test set varied from 5 to 9,950 nmol/L. The test set compounds were classified as active, moderately active, and inactive based on their IC_50_ values, similar to training set compounds. **Table S2** shows that most of the compounds were anticipated to be in the same range as their experimental range, except for three moderately active compounds that were overestimated active. Furthermore, Hypo1 reflects a significant correlation between the predicted and actual biological activities of the training set (R^2^ = 0.98) and test set (R^2^ = 0.91) (**Figure 5**). The Hypo1 validation results indicate that the selected hypothesis fulfilled the criteria suggested by previous reports to be an ideal pharmacophore model for virtual screening of drug-like databases (Sakkiah and Lee, 2012; Kumar et al., 2015).

### 3.3 Virtual Screening of the ZINC Database

A compound’s drug-like properties are a prerequisite for it to be utilized as a potential drug molecule. Therefore, the ZINC database embedded with 144,766 natural products was filtered *via* Ro5 and Veber’s rules, resulting in 98,842 compounds (Lipinski et al., 2001; Veber et al., 2002). This huge number of compounds was further filtered *via* ADMET pharmacokinetic criteria, resulting in 7,378 drug-like natural compounds. Subsequently, the well-validated 3D-QSAR model Hypo1 was employed as a 3D query to screen natural compounds using the *Ligand Pharmacophore Mapping* function of DS. The mapping yielded 2,964 drug-like molecules that mapped every aspect of the model. Lastly, the obtained compounds were manually scrutinized for false-positive compounds mapping the model. This process resulted in 2,277 actual positive compounds, which were proceeded for molecular docking with the SYK structure.

### 3.4 Molecular Docking

The molecules acquired from the pharmacophore-based screening were docked with the catalytic site of the SYK domain structure (PDB ID: 6VOV) (Blomgren et al., 2020). The reliability of GOLD docking software was evaluated before the docking of drug-like molecules (Kumar et al., 2021b). In this process, the re-docking of co-crystallized ligand lanraplenib was performed, resulting in a root mean square deviation (RMSD) of <2 Å among the docked and co-crystallized poses (**Figure S2A**). Consequently, the docking-based virtual screening of 2,277 mapped compounds was executed with the generation of one conformer per ligand. A total of 129 compounds exhibited higher GoldScores and lower ChemScores than the REF inhibitors lanraplenib and fostamatinib selected initially for detailed molecular interaction analysis (Kumar et al., 2021b; Parate et al., 2021b). We performed a more extensive docking evaluation for the 129 molecules, with 10 conformers per ligand generation. Our docking analyses revealed that lanraplenib showed a GoldScore of 64.56 and ChemScore of -28.99, while fostamatinib demonstrated GoldScore of 66.62 and ChemScore -17.60. Using reference inhibitors’ docking scores as cutoff, 33 compounds were selected (**Figure S2B**). It is noteworthy to mention that selected compounds displayed significantly better docking scores when compared with REF drugs. For example, top-ranked docked compound ZINC98364146 displayed a GoldScore of 77.50 and a ChemScore of -34.12, which is much better than the REF compounds’ score. **Table S3** displays the complete analysis of each compound’s docking score and hydrogen-bond details. Our structural observation of 62 SYK inhibitor-bound PDB structures reveals that Ala451 and Asp512 were targeted *via* hydrogen bond by 59 and 25 inhibitors, respectively. Interestingly, the selected docked compound’s hydrogen-bond analysis shows that each compound targets key residue Ala451 or Asp512 *via* a hydrogen bond (**Table S3**). Moreover, previous docking studies with the SYK protein revealed that active site residues such as Leu377, Ser379, Val385, Met448, Ala451, Lys458, Asp499, and Asp512 were actively observed during protein–ligand interactions; a similar observation during our analysis supports our selection (Huang et al., 2017; Wang et al., 2018). Finally, 33 selected compounds were forward for molecular simulations to monitor their stability under a virtual cellular environment.

### 3.5 Molecular Dynamics Simulations

The MD simulations are a broadly used method to study the steadiness of protein–ligand systems at the atomic level (Karplus and McCammon, 2002). The stability of the system was calculated using geometric features such as root mean square deviation (RMSD) and root mean square fluctuations (RMSF) (Kumar et al., 2021a). Further, the computationally rigorous method MM-PBSA was utilized to screen the simulated complexes (Kumari et al., 2014; Kumar et al., 2021c). The binding free energy of the REF inhibitors lanraplenib (REF1) and fostamatinib (REF2) was used as the first criteria for selecting the potential hit compounds among 33 simulated inhibitor–SYK complexes (**Table S3**) (Kumar et al., 2021d; Parate et al., 2021c). The selected hit compounds were analyzed in detail and are discussed below.

#### 3.5.1 Stability of the Simulated Systems

**Figure 6:**
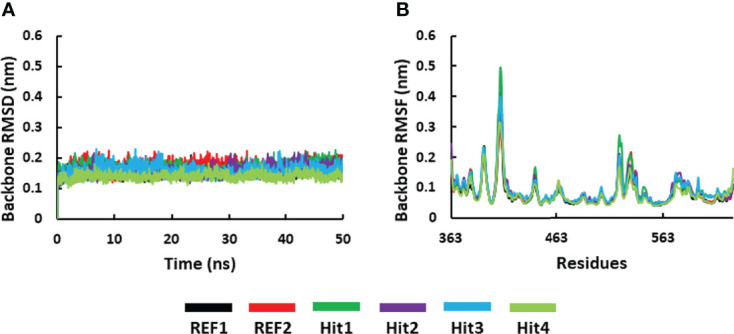
Molecular dynamics simulation analysis. **(A)** RMSD and **(B)** the RMSF plot for the backbone atoms of the SYK protein. The graphs were calculated for 50 ns of the simulation run.

The RMSD of SYK protein backbone atoms was calculated to determine the stability of all the simulated systems throughout the simulation run. It is shown in **Figure 6A** that all the systems displayed stable behavior from start to endpoint. The average RMSD value for all the systems was observed below 0.3 nm, which indicated that simulated complexes displayed RMSD values below the threshold. The average RMSD values further showed that the Hit4–SYK complex displayed less deviation (0.14 nm), whereas Hit2 and Hit3 demonstrated a similar average value to REF1 (0.15 nm). Further, Hit1 and REF2 showed slightly high average RMSD values of 0.16 and 0.17 nm, respectively. RMSF is an important variable used to display each residue’s fluctuation rate upon ligand binding. **Figure 6B** shows the RMSF plot for all the simulated systems. It can be seen from the RMSF plot that all the simulated complexes displayed a similar pattern of fluctuations at residues 408–410, indicating significant movement of protein backbone atoms; these residues are from the loop region of the protein and are not part of the active site region. Furthermore, it was observed that none of the active site residues displayed an RMSF value >0.3 nm. This indicates that active site residues were minimally disturbed upon binding of the ligand or known drug.

#### 3.5.2 Binding Free Energy Analysis

**Figure 7:**
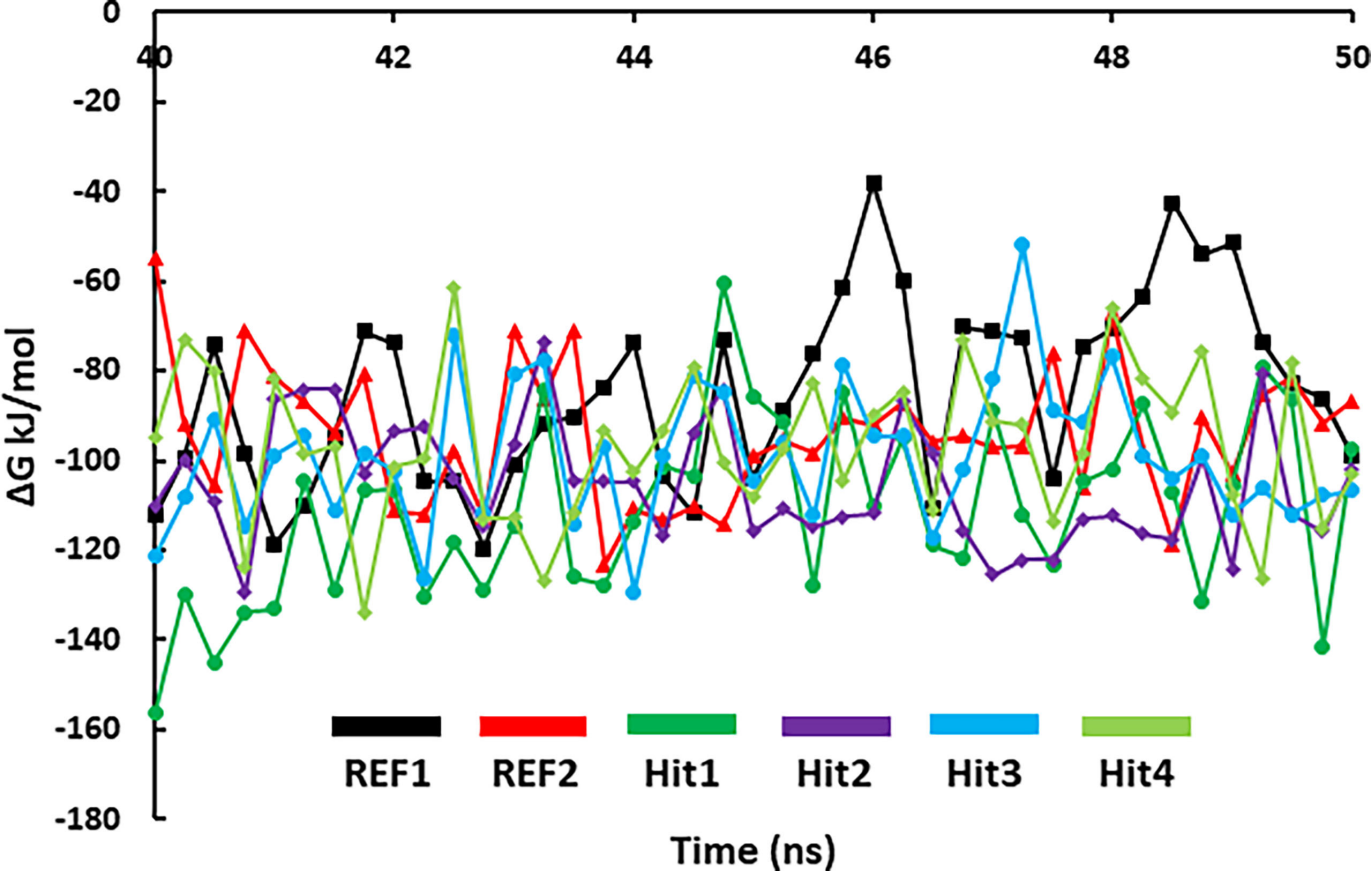
MM/PBSA predicted binding free energy of SYK-bound hit compounds and REF inhibitors. The stable trajectories from the last 10-ns MD simulations were utilized to estimate the energy values.

MD simulations collective with binding free-energy calculations were used to accurately estimate the binding affinity of drug-like molecules (**Table S3**). The MM-PBSA scoring typically correlates with experimentally determined activity (Genheden and Ryde, 2015). **Table 3** demonstrates the results of the binding free energy prediction of the REF inhibitors and selected compounds. The REF inhibitor fostamatinib and co-crystal drug lanraplenib displayed binding free energy values of -90.00 and -84.53 kJ/mol, respectively. In contrast, the potential hit compounds showed lower binding free energy values than REF compounds (**Table 3** and **Figure 7**). The lowest free energy value is indicative of the strong binding affinity with protein; therefore, we ranked our hit compounds according to free energy values. Hit1 displayed an average binding free energy value of -111.11 kJ/mol, followed by Hit2, Hit3, and Hit4 with values of -105.30, -98.81, and -96.76 kJ/mol, respectively. The energy decomposition was further performed to understand the energetics of all the systems. **Table 3** indicates that van der Waals, electrostatic, and SASA energy components energetically favored the formation of the protein–ligand complex, whereas polar solvation opposed the formation of the complex. Free energy data further reveal that van der Waals interactions contribute pointedly to the protein–ligand complexes trailed by electrostatic and SASA energy.

**Table 3:** The detailed distribution of the binding free energy factors calculated from MM-PBSA method for hit and REF compounds.

Inhibitors	van der Waals (kJ/mol)	Electrostatic (kJ/mol)	Polar solvation (kJ/mol)	SASA energy (kJ/mol)	Binding energy ΔG_bind_ (kJ/mol)
**Hit1**	-210.95+/-10.15	-58.93+/-16.98	180.96+/-32.26	-22.19+/-1.10	-111.11+/-20.13
**Hit2**	-200.49+/-15.31	-27.23+/-7.95	143.73+/-16.07	-21.31+/-1.54	-105.30+/-13.45
**Hit3**	-190.14+/-11.53	-95.23+/-29.87	206.86+/-31.84	-20.29+/-1.43	-98.81+/-15.54
**Hit4**	-212.89+/-10.85	-50.55+/-13.19	188.66+/-21.25	-21.98+/-1.23	-96.76+/-16.80
**REF1**	-181.03+/-9.33	-63.90+/-15.32	178.61+/-33.69	-18.21+/-1.08	-84.53+/-20.67
**REF2**	-210.76+/-11.57	-65.89+/-8.95	203.25+/-35.50	-20.59+/-1.18	-93.94+/-32.01

REF1, lanraplenib; REF2, fostamatinib.

#### 3.5.3 Binding Mode Analysis

**Figure 8:**
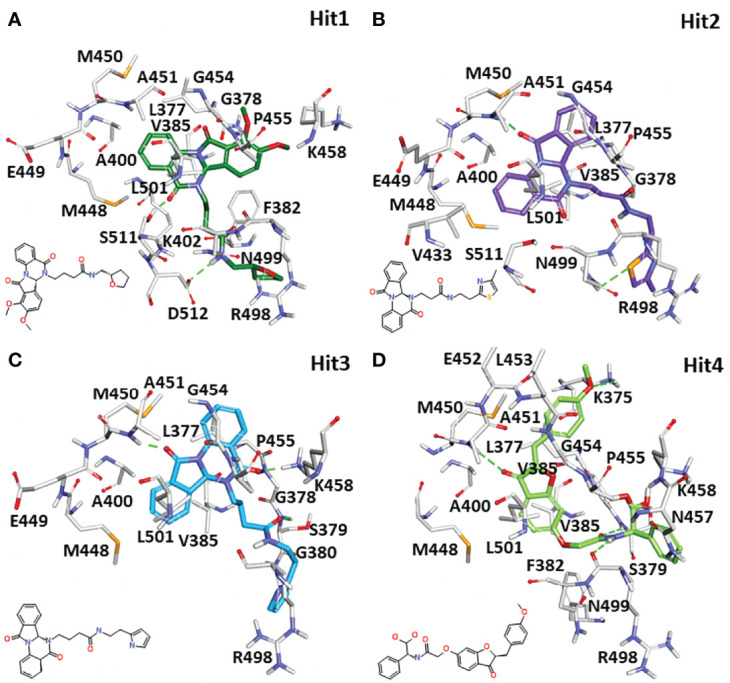
The 3D molecular interactions of the designated hit compounds inside the ATP competitive site of SYK. **(A)** Hit1, **(B)** Hit2, **(C)** Hit3, and **(D)** Hit4 are shown with the different color schemes in stick representation. The protein in the background is shown with a gray color line representation. The protein residues involved in polar and non-polar interactions were shown in gray stick representations. The hydrogen bonds were shown with green dashed lines. The 2D structure of each hit was shown on the left side of each 3D interaction image.

Identifying the molecular interaction of the potential inhibitors with the target protein is the key finding of the ligand and structure-based drug discovery reports. Lee et al. demonstrated that the SYK ATP-binding site can be divided into four subsites: the hinge region, the glycine-rich loop, the DFG motif, and the activation loop (**Figure 3**) (Lee et al., 2016). The average structures of complexes calculated from MD simulations were superimposed to study the binding mode. The superimposition of all individual complexes confirms that each inhibitor lodged in the ATP-binding site of SYK and obtained an analogous orientation to the co-crystal ligand (**Figure S3**). A literature survey revealed that gatekeeper Met448, hinge region Ala451 and Pro455, C-terminal Arg498, and DFG motif residue Asp512 are crucial for the interaction of inhibitors. Additionally, Pro455 is reported to provide selectivity for the inhibitors (Lucas et al., 2012; Lucas et al., 2014). The average structure of SYK bound with lanraplenib displayed two hydrogen bonds with Ala451 and Ser379 (**Figure S4A**). The binding mode of the lanraplenib-simulated SYK complex followed the X-ray structure of SYK bound with lanraplenib where a hydrogen bond with Ala451 was observed (Blomgren et al., 2020). Fostamatinib was noticed to show hydrogen bond interactions with Leu377, Glu376, and Ala451 (**Figure S4B**). The simulation studies were not reported in the past for fostamatinib with the SYK protein, but interestingly its prodrug R406 was studied under simulation conditions, and it was reported that it could form hydrogen bond interactions with Leu377 and Ala451, similar to our predicted binding mode (Marchetti et al., 2020). The molecular interactions of both the REF inhibitors were strengthened by multiple hydrophobic interactions with SYK active site residues (**Figures S4C**, **S4D**, and **Table 4**). The binding mode of the selected SYK inhibitors was observed to follow the REF inhibitors, especially the co-crystal drug lanraplenib. The binding mode of fostamatinib may be slightly different from other compounds. Interestingly Hit1, Hit2, and Hit3 are observed to have a quinazoline ring, and surprisingly Hit4 was observed to have a benzofuran ring (**Table S4**). Numerous reports suggest that both scaffolds have strong anticancer, antimicrobial, and antiviral effects; therefore, it can be hypothesized that these compounds may show good inhibitory potential in experimental studies (Nevagi et al., 2015; Bhat et al., 2021). The detailed molecular interactions of identified hit compounds are exposed in **Figure 8**. It can be perceived from **Figure 8A** that Hit1 forms hydrogen bond interactions with Arg498, Ser511, and DFG motif residue Asp512. Despite having similar scaffolds, Hit2 and Hit3 target different active site residues through hydrogen bonds. Hit2 forms a hydrogen bond interaction with Ala451 and Asn499 (**Figure 8B**). Hit3 forms hydrogen bonds with Ser379, Lys458, and Ala451 (**Figure 8C**). However, Hit4 obtained from different scaffold forms hydrogen bond interactions with Ser379, Lys375, Ala451, and Arg498 (**Figures 8D**). The quinazoline ring of Hit1, Hit2, and Hit3 was involved in multiple π–alkyl interactions, while this interaction was formed by the benzofuran ring in Hit4. Overall, it was observed that hit compounds similarly bind ATP-binding sites as REF inhibitors and target key active site residues. As inhibitors identified in the present study are of type I class, it is essential to compare the molecular interactions of hits with crucial residues of the ATP competitive site of SYK to achieve high selectivity over other kinases (Gagic et al.). Previous studies indicated that the sequence alignment of multiple kinases reveals residues Pro455 and Asn457 are the rare combination in the active site of SYK. Therefore, the inhibitor interaction with these residues may provide high selectivity to the compounds (Lucas et al., 2012; Huang et al., 2017). The detailed 2D molecular interaction maps of Hit1, Hit2, Hit3, and Hit4 showed that all form hydrophobic interactions with unique residue Pro455, and more interestingly, Hit4 also includes interactions with Asn457 (**Figures S5A–D**). Furthermore, a recent study pointed out that direct interactions with Asn499 may lead to a high selectivity for SYK inhibition (Lee et al., 2016). It is noteworthy that Hit2 forms a hydrogen bond with Asn499, whereas Hit1, Hit3, and Hit4 displayed a van der Waals interaction with Asn499. The detailed molecular interaction hydrogen bond distances of Hit1, Hit2, Hit3, Hit4, REF1, and REF2 are shown in **Table 4**, and **Figures S4**-**S5**.

**Table 4:** The detailed molecular interactions of the potential SYK inhibitors and REF drugs with its active site residues.

Sr. no.	Hydrogen bond interactions				van der Waals interactions	π–π/π-alkyl interactions
	Amino acid	Amino acid atom	Ligand atom	Distance (<3.0 Å)		
**Hit1**	Arg498	HH11	O32	2.39	Gly378, Phe382, Lys402, Glu449, Met450, Gly454, Pro455, Lys458, Asn499	Leu377, Val385, Ala400, Met448, Ala451, Leu501
	Ser511	HG1	O1	1.94
	Asp512	OD2	H49	2.22
**Hit2**	Ala451	HN	O28	1.99	Gly378, Val385, Glu449, Met450, Gly454, Pro455, Ser511	Leu377, Ala400, Val433, Met448, Arg498, Leu501
	Asn499	HD22	S1	2.90
**Hit3**	Ser379	HN	O24	2.92	Gly378, Gly380, Glu449, Met450, Gly454, Arg498	Leu377, Val385, Ala400, Pro455, Met448, Leu501
	Ala451	HN	O19	1.99
	Lys458	HZ1	O1	1.69
**Hit4**	Lys375	HZ1	O21	2.79	Gly378, Phe382, Ala400, Met450, Glu452, Leu453, Gly454, Pro455, Asn457, Lys458, Asn499	Leu377, Val385, Met448, Leu501
	Ser379	HN	O22	2.54
	Ala451	HN	O1	2.65
	Arg498	O	H45	2.33
**REF1**	Ser379	O	H58	1.99	Gly380, Phe382, Lys402, Glu449, Met450, Glu452, Gly454, Pro455, Lys458, Asn499	Gly378, Val385, Ala400, Met448, Leu453, Leu501
	Ala451	HN	N7	2.13
	Ala451	O	H51	2.29
**REF2**	Glu376	O	H52	2.76	Gly378, Phe382, Ala400, Met450, Glu452, Gly454, Pro455, Asn499	Lys375, Val385, Lys458, Arg498, Leu501
	Leu377	O	H51	2.49
	Ala451	O	H55	2.87
	Ala451	O	H56	1.68
	Ala451	HN	O8	2.02

REF1, lanraplenib; REF2, fostamatinib.

#### 3.5.4 Per-Residue Energy Decomposition Analysis

**Figure 9:**
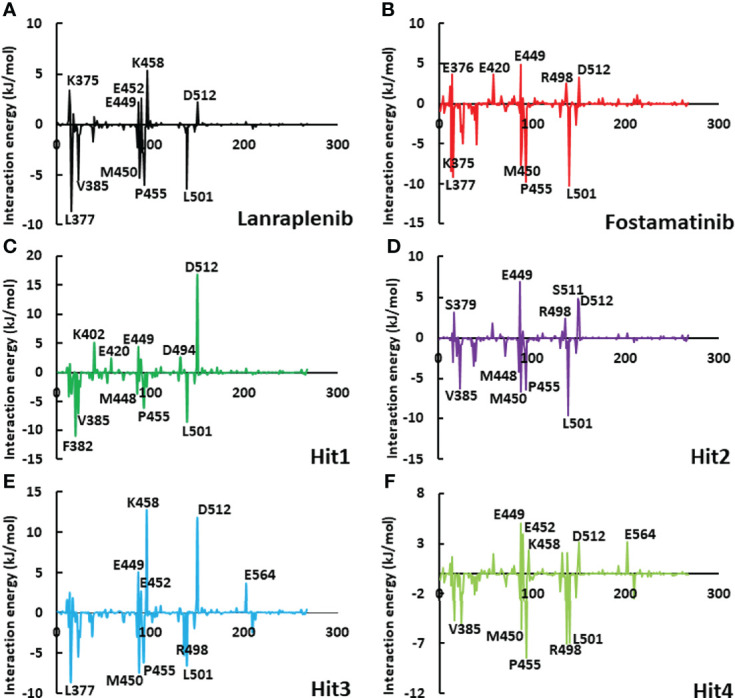
The graphical plot of the binding free energy decomposition on per residue for SYK-inhibitor complexes. The individual plots of **(A)** lanraplenib, **(B)** fostamatinib, **(C)** Hit1, **(D)** Hit2, **(E)** Hit3, and **(F)** Hit4 were shown with different color schemes. The residues shown on the graph’s upper side could contribute to electrostatic interactions, whereas residues on the lower side of the graph could contribute to hydrophobic interactions.

To get a detailed description of the contribution for each hotspot residue, the per-residue contribution was determined using the MM-PBSA method (Parate et al., 2021a). **Figure 9** highlights the per-residue contribution where the top five residues involved in polar and non-polar interactions were shown. The active site residues Leu377, Phe382, Val385, Met488, Met450, Pro455, Arg498, and Leu501 significantly contributed to binding lanraplenib, fostamatinib, and hit compounds. Moreover, in the case of lanraplenib, residue Leu377 displayed the highest contribution for binding -8.62 kJ/mol, whereas, for fostamatinib, it was observed to be Leu501 (-10 kJ/mol). The highest contributing residue for binding of hit compounds was Phe382 (-10 kJ/mol) for Hit1, Leu501 (-10 kJ/mol) for Hit2, Leu377 (-8.51 kJ/mol) for Hit3, and Leu377 (-10 kJ/mol) for Hit4. Interestingly, the key residue Pro455 was reported to bind all the hit compounds, but a significant contribution was observed for Hit4 (-8.44 kJ/mol). The contribution by Pro455 for REF inhibitors was observed more significantly in fostamatinib (-9.76 kJ/mol) than lanraplenib (-4.5 kJ/mol). The residues Lys375, Ser379, Glu376, Lys402, Glu420, Glu449, Glu452, Lys458, Asp494, Arg498, Asp512, and Glu564 were reported to contribute positively; therefore, they may be responsible for polar interactions in REF inhibitors and hit compounds. From this analysis, it can be concluded that in non-polar interactions Pro455 and Leu501 were observed in both the REF inhibitors as well as all four selected hit compounds, indicating their significant role in inhibitor engagement; moreover, for polar interaction, Glu449 and Asp512 may be critical. This analysis provides essential information about the role of each active site residue in inhibitor binding and can thus be exploited in future structure-guided SYK inhibition studies.

## 4 Conclusion

In the present report, we have produced a 3D-QSAR pharmacophore model for SYK utilizing the bioactivity knowledge of established inhibitors. The key features required for SYK inhibition were ensembled in a 3D model and further employed to screen a drug-like database to identify novel SYK inhibitor scaffolds. The affinity of the potential inhibitors toward SYK was studied using integrated molecular docking and molecular dynamics simulations. The two REF inhibitors, fostamatinib and lanraplenib, were used to select the final hit compounds based on key molecular interactions and lower binding free energy values. We propose four novel scaffolds which show desirable interactions with residues reported crucial for SYK inhibition as virtual candidates. These SYK inhibitor scaffolds with good drug-like properties may be utilized for SYK inhibition programs against various autoimmune and inflammatory diseases.

## Data Availability Statement

The original contributions presented in the study are included in the article/**Supplementary Material**. Further inquiries can be directed to the corresponding author.

## Author Contributions

VK and KL designed the study. VK and SP performed the experiments. VK compiled the manuscript. VK, SP, and KL analyzed the results. D and AZ did the formal analysis. All authors contributed to the article and approved the submitted version.

## Funding

This work was supported by two National Research Foundation of Korea (NRF) grants funded by the Korean government (MSIT) (Grant Nos. NRF-2021R1F1A1059797 and NRF-2021R1A2B5B02002220).

## Conflict of Interest

The authors declare that the research was conducted in the absence of any commercial or financial relationships that could be construed as a potential conflict of interest.

## Publisher’s Note

All claims expressed in this article are solely those of the authors and do not necessarily represent those of their affiliated organizations, or those of the publisher, the editors and the reviewers. Any product that may be evaluated in this article, or claim that may be made by its manufacturer, is not guaranteed or endorsed by the publisher.

## References

[B1] Bender B. J. Gahbauer S. Luttens A. Lyu J. Webb C. M. Stein R. M.. (2021). A Practical Guide to Large-Scale Docking. Nat. Protoc. 2021 1610 16, 4799–4832. doi: 10.1038/s41596-021-00597-z PMC8522653 34561691

[B2] Bhat M. Belagali S. L. Mamatha S. V. Sagar B. K. Sekhar E. V. (2021). Importance of Quinazoline and Quinazolinone Derivatives in Medicinal Chemistry. Stud. Nat. Prod. Chem. 71, 185–219. doi: 10.1016/B978-0-323-91095-8.00005-2

[B3] Blomgren P. Chandrasekhar J. Di Paolo J. A. Fung W. Geng G. Ip C.. (2020). Discovery of Lanraplenib (GS-9876): A Once-Daily Spleen Tyrosine Kinase Inhibitor for Autoimmune Diseases. ACS Med. Chem. Lett. 11, 506–513. doi: 10.1021/ACSMEDCHEMLETT.9B00621/SUPPL_FILE/ML9B00621_SI_001.PDF 32292557 PMC7153012

[B4] Cheng A. M. Rowley B. Pao W. Hayday A. Bolen J. B. Pawson T. (1995). Syk Tyrosine Kinase Required for Mouse Viability and B-Cell Development. Nature 378, 303–306. doi: 10.1038/378303A0 7477353

[B5] Currie K. S. Kropf J. E. Lee T. Blomgren P. Xu J. Zhao Z.. (2014). Discovery of GS-9973, a Selective and Orally Efficacious Inhibitor of Spleen Tyrosine Kinase. J. Med. Chem. 57, 3856–3873. doi: 10.1021/JM500228A/SUPPL_FILE/JM500228A_SI_001.PDF 24779514

[B6] Debnath A. K. (2002). Pharmacophore Mapping of a Series of 2,4-Diamino-5-Deazapteridine Inhibitors of Mycobacterium Avium Complex Dihydrofolate Reductase. J. Med. Chem. 45, 41–53. doi: 10.1021/JM010360C 11754578

[B7] Duong-Ly K. C. Peterson J. R. (2013). The Human Kinome and Kinase Inhibition as a Therapeutic Strategy. Curr. Protoc. Pharmacol. 0 2. doi: 10.1002/0471141755.PH0209S60 PMC4128285 23456613

[B8] Farmer L. J. Bemis G. Britt S. D. Cochran J. Connors M. Harrington E. M.. (2008). Discovery and SAR of Novel 4-Thiazolyl-2-Phenylaminopyrimidines as Potent Inhibitors of Spleen Tyrosine Kinase (SYK). Bioorg. Med. Chem. Lett. 18, 6231–6235. doi: 10.1016/J.BMCL.2008.09.106 18938080

[B9] Gagic Z. Ruzic D. Djokovic N. Djikic T. Nikolic K.. (2020). *In Silico* Methods for Design of Kinase Inhibitors as Anticancer Drugs. Front. Chem. 7. doi: 10.3389/fchem.2019.00873 PMC6960140 31970149

[B10] Genheden S. Ryde U. (2015). The MM/PBSA and MM/GBSA Methods to Estimate Ligand-Binding Affinities. Expert Opin. Drug Discovery 10, 449. doi: 10.1517/17460441.2015.1032936 PMC4487606 25835573

[B11] Graves J. D. Krebs E. G. (1999). Protein Phosphorylation and Signal Transduction. Pharmacol. Ther. 82, 111–121. doi: 10.1016/S0163-7258(98)00056-4 10454190

[B12] Hirabayashi A. Mukaiyama H. Kobayashi H. Shiohara H. Nakayama S. Ozawa M.. (2008). Structure-Activity Relationship Studies of Imidazo[1,2-C]Pyrimidine Derivatives as Potent and Orally Effective Syk Family Kinases Inhibitors. Bioorg. Med. Chem. 16, 9247–9260. doi: 10.1016/J.BMC.2008.09.015 18823784

[B13] Hong Y. H. Kim J. H. Cho J. Y. (2020). Ranunculus Bulumei Methanol Extract Exerts Anti-Inflammatory Activity by Targeting Src/Syk in NF-κb Signaling. Biomolecules 10. doi: 10.3390/BIOM10040546 PMC7226355 32260181

[B14] Huang Y. Zhang Y. Fan K. Dong G. Li B. Zhang W.. (2017). Discovery of New Syk Inhibitors Through Structure-Based Virtual Screening. Bioorg. Med. Chem. Lett. 27, 1776–1779. doi: 10.1016/J.BMCL.2017.02.060 28268139

[B15] Humphrey W. Dalke A. Schulten K. (1996). VMD: Visual Molecular Dynamics. J. Mol. Graph. 14, 33–38. doi: 10.1016/0263-7855(96)00018-5 8744570

[B16] John S. Thangapandian S. Arooj M. Hong J. C. Kim K. D. Lee K. W. (2011). Development, Evaluation and Application of 3D QSAR Pharmacophore Model in the Discovery of Potential Human Renin Inhibitors. BMC Bioinf. 12 Suppl 14. doi: 10.1186/1471-2105-12-S14-S4 PMC3287469 22372967

[B17] Jones G. Willett P. Glen R. C. Leach A. R. Taylor R. (1997). Development and Validation of a Genetic Algorithm for Flexible Docking. J. Mol. Biol. 267, 727–748. doi: 10.1006/JMBI.1996.0897 9126849

[B18] Karplus M. McCammon J. A. (2002). Molecular Dynamics Simulations of Biomolecules. Nat. Struct. Biol. 9, 646–652. doi: 10.1038/nsb0902-646 12198485

[B19] Kaserer T. Beck K. R. Akram M. Odermatt A. Schuster D. Willett P. (2015). Pharmacophore Models and Pharmacophore-Based Virtual Screening: Concepts and Applications Exemplified on Hydroxysteroid Dehydrogenases. Molecules 20, 22799. doi: 10.3390/MOLECULES201219880 26703541 PMC6332202

[B20] Kaur M. Singh M. Silakari O. (2013). Inhibitors of Switch Kinase “Spleen Tyrosine Kinase” in Inflammation and Immune-Mediated Disorders: A Review. Eur. J. Med. Chem. 67, 434–446. doi: 10.1016/J.EJMECH.2013.04.070 23917087

[B21] Kobayashi T. Nakamura S. Taniguchi T. Yamamura H. (1990). Purification and Characterization of a Cytosolic Protein-Tyrosine Kinase From Porcine Spleen. Eur. J. Biochem. 188, 535–540. doi: 10.1111/J.1432-1033.1990.TB15433.X 2331984

[B22] Kollman P. A. Massova I. Reyes C. Kuhn B. Huo S. Chong L.. (2000). Calculating Structures and Free Energies of Complex Molecules: Combining Molecular Mechanics and Continuum Models. Acc. Chem. Res. 33, 889–897. doi: 10.1021/AR000033J 11123888

[B23] Kumari R. Kumar R. Lynn A. (2014). G-Mmpbsa -A GROMACS Tool for High-Throughput MM-PBSA Calculations. J. Chem. Inf. Model. 54, 1951–1962. doi: 10.1021/CI500020M/SUPPL_FILE/CI500020M_SI_001.PDF 24850022

[B24] Kumar R. Kumar V. Lee K. W. (2021a). A Computational Drug Repurposing Approach in Identifying the Cephalosporin Antibiotic and Anti-Hepatitis C Drug Derivatives for COVID-19 Treatment. Comput. Biol. Med. 130, 104186. doi: 10.1016/J.COMPBIOMED.2020.104186 33360831 PMC7748973

[B25] Kumar V. Kumar R. Parate S. Yoon S. Lee G. Kim D.. (2021b). Identification of ACK1 Inhibitors as Anticancer Agents by Using Computer-Aided Drug Designing. J. Mol. Struct. 1235, 130200. doi: 10.1016/J.MOLSTRUC.2021.130200

[B26] Kumar V. Parate S. Thakur G. Lee G. Ro H. S. Kim Y.. (2021c). Identification of CDK7 Inhibitors From Natural Sources Using Pharmacoinformatics and Molecular Dynamics Simulations. Biomed 9 1197. doi: 10.3390/BIOMEDICINES9091197 PMC8468199 34572383

[B27] Kumar V. Parate S. Yoon S. Lee G. Lee K. W. (2021d). Computational Simulations Identified Marine-Derived Natural Bioactive Compounds as Replication Inhibitors of SARS-CoV-2. Front. Microbiol. 12. doi: 10.3389/FMICB.2021.647295/BIBTEX PMC8097174 33967984

[B28] Kumar R. Son M. Bavi R. Lee Y. Park C. Arulalapperumal V.. (2015). Novel Chemical Scaffolds of the Tumor Marker AKR1B10 Inhibitors Discovered by 3D QSAR Pharmacophore Modeling. Acta Pharmacol. Sin. 36, 998–1012. doi: 10.1038/APS.2015.17 26051108 PMC4564875

[B29] Lee S. J. Choi J. S. Han B. G. Kim H. S. Song H. J. Lee J.. (2016). Crystal Structures of Spleen Tyrosine Kinase in Complex With Novel Inhibitors: Structural Insights for Design of Anticancer Drugs. FEBS J. 283, 3613–3625. doi: 10.1111/FEBS.13831 27504936

[B30] Liddle J. Atkinson F. L. Barker M. D. Carter P. S. Curtis N. R. Davis R. P.. (2011). Discovery of GSK143, a Highly Potent, Selective and Orally Efficacious Spleen Tyrosine Kinase Inhibitor. Bioorg. Med. Chem. Lett. 21, 6188–6194. doi: 10.1016/J.BMCL.2011.07.082 21903390

[B31] Lipinski C. A. Lombardo F. Dominy B. W. Feeney P. J. (2001). Experimental and Computational Approaches to Estimate Solubility and Permeability in Drug Discovery and Development Settings. Adv. Drug Deliv. Rev. 46, 3–26. doi: 10.1016/S0169-409X(00)00129-0 11259830

[B32] Liu D. Mamorska-Dyga A. (2017). Syk Inhibitors in Clinical Development for Hematological Malignancies. J. Hematol. Oncol. 10, 1–7. doi: 10.1186/S13045-017-0512-1/TABLES/1 28754125 PMC5534090

[B33] Lucas M. C. Bhagirath N. Chiao E. Goldstein D. M. Hermann J. C. Hsu P. Y.. (2014). Using Ovality to Predict Nonmutagenic, Orally Efficacious Pyridazine Amides as Cell Specific Spleen Tyrosine Kinase Inhibitors. J. Med. Chem. 57, 2683–2691. doi: 10.1021/JM401982J/SUPPL_FILE/JM401982J_SI_001.PDF 24520947

[B34] Lucas M. C. Goldstein D. M. Hermann J. C. Kuglstatter A. Liu W. Luk K. C.. (2012). Rational Design of Highly Selective Spleen Tyrosine Kinase Inhibitors. J. Med. Chem. 55, 10414–10423. doi: 10.1021/JM301367C/SUPPL_FILE/JM301367C_SI_001.PDF 23151054

[B35] Mali S. N. Pandey A. (2021). Molecular Modeling Studies on 2,4-Disubstituted Imidazopyridines as Anti-Malarials: Atom-Based 3d-QSAR, Molecular Docking, Virtual Screening, *In-Silico* ADMET and Theoretical Analysis. J. Comput. Biophys. Chem. 20, 267–282. doi: 10.1142/S2737416521500125

[B36] Mali S. N. Pandey A. (2022). Balanced QSAR and Molecular Modeling to Identify Structural Requirements of Imidazopyridine Analogues as Anti-Infective Agents Against Trypanosomiases. J. Comput. Biophys. Chem. 21, 83–114. doi: 10.1142/S2737416521410015

[B37] Marchetti G. Dessì A. Dallocchio R. Tsamesidis I. Pau M. C. Turrini F. M.. (2020). Syk Inhibitors: New Computational Insights Into Their Intraerythrocytic Action in Plasmodium Falciparum Malaria. Int. J. Mol. Sci. 21 7009. doi: 10.3390/IJMS21197009 PMC7582821 32977621

[B38] Mócsai A. Ruland J. Tybulewicz V. L. J. (2010). The SYK Tyrosine Kinase: A Crucial Player in Diverse Biological Functions. Nat. Rev. Immunol. 10, 387–402. doi: 10.1038/nri2765 20467426 PMC4782221

[B39] Mullard A. (2018). FDA Approves First-in-Class SYK Inhibitor. Nat. Rev. Drug Discovery 17, 385. doi: 10.1038/NRD.2018.96 29844593

[B40] Nam S. T. Kim H. W. Kim H. S. Park Y. H. Lee D. Lee M. B.. (2018). Furaltadone Suppresses IgE-Mediated Allergic Response Through the Inhibition of Lyn/Syk Pathway in Mast Cells. Eur. J. Pharmacol. 828, 119–125. doi: 10.1016/J.EJPHAR.2018.03.035 29588153

[B41] Nevagi R. J. Dighe S. N. Dighe S. N. (2015). Biological and Medicinal Significance of Benzofuran. Eur. J. Med. Chem. 97, 561–581. doi: 10.1016/J.EJMECH.2014.10.085 26015069

[B42] Padilla F. Bhagirath N. Chen S. Chiao E. Goldstein D. M. Hermann J. C.. (2013). Pyrrolopyrazines as Selective Spleen Tyrosine Kinase Inhibitors. J. Med. Chem. 56, 1677–1692. doi: 10.1021/JM301720P/SUPPL_FILE/JM301720P_SI_001.PDF 23350847

[B43] Pamuk O. N. Tsokos G. C. (2010). Spleen Tyrosine Kinase Inhibition in the Treatment of Autoimmune, Allergic and Autoinflammatory Diseases. Arthritis Res. Ther. 12, 1–11. doi: 10.1186/AR3198/FIGURES/6 PMC3046528 21211067

[B44] Parate S. Kumar V. Chan Hong J. Lee K. W. (2021a). Investigating Natural Compounds Against Oncogenic RET Tyrosine Kinase Using Pharmacoinformatic Approaches for Cancer Therapeutics. RSC Adv. 12, 1194–1207. doi: 10.1039/D1RA07328A 35425116 PMC8978841

[B45] Parate S. Kumar V. Danishuddin Hong J. C. Lee K. W. (2021b). Computational Investigation Identified Potential Chemical Scaffolds for Heparanase as Anticancer Therapeutics. Int. J. Mol. Sci. 22. doi: 10.3390/IJMS22105311 PMC8157885 34156395

[B46] Parate S. Kumar V. Hong J. C. Lee K. W. (2021c). Investigation of Marine-Derived Natural Products as Raf Kinase Inhibitory Protein (RKIP)-Binding Ligands. Mar. Drugs 19, 581. doi: 10.3390/MD19100581 34677480 PMC8539980

[B47] Pronk S. Páll S. Schulz R. Larsson P. Bjelkmar P. Apostolov R.. (2013). GROMACS 4.5: A High-Throughput and Highly Parallel Open Source Molecular Simulation Toolkit. Bioinformatics 29, 845–854. doi: 10.1093/BIOINFORMATICS/BTT055 23407358 PMC3605599

[B48] Sakkiah S. Lee K. W. (2012). Pharmacophore-Based Virtual Screening and Density Functional Theory Approach to Identifying Novel Butyrylcholinesterase Inhibitors. Acta Pharmacol. Sin. 33, 964–978. doi: 10.1038/APS.2012.21 22684028 PMC4077067

[B49] Sakkiah S. Thangapandian S. John S. Kwon Y. J. Lee K. W. (2010). 3d QSAR Pharmacophore Based Virtual Screening and Molecular Docking for Identification of Potential HSP90 Inhibitors. Eur. J. Med. Chem. 45, 2132–2140. doi: 10.1016/J.EJMECH.2010.01.016 20206418

[B50] Sapay N. Tieleman D. P. (2011). Combination of the CHARMM27 Force Field With United-Atom Lipid Force Fields. J. Comput. Chem. 32, 1400–1410. doi: 10.1002/JCC.21726 21425293

[B51] Shao Y. Zhang S. Zhang Y. Liu Z. (2021). Recent Advance of Spleen Tyrosine Kinase in Diseases and Drugs. Int. Immunopharmacol. 90, 107168. doi: 10.1016/J.INTIMP.2020.107168 33264719

[B52] Siveen K. S. Prabhu K. S. Achkar I. W. Kuttikrishnan S. Shyam S. Khan A. Q.. (2018). Role of Non Receptor Tyrosine Kinases in Hematological Malignances and its Targeting by Natural Products. Mol. Cancer 17. doi: 10.1186/S12943-018-0788-Y PMC5817858 29455667

[B53] Sundarapandian T. Shalini J. Sugunadevi S. Woo L. K. (2010). Docking-Enabled Pharmacophore Model for Histone Deacetylase 8 Inhibitors and its Application in Anti-Cancer Drug Discovery. J. Mol. Graph. Model. 29, 382–395. doi: 10.1016/J.JMGM.2010.07.007 20870437

[B54] Taniguchis,. T. Kobayashiss T. Kondon J. Takahashill K. Nakamurall H. Suzukill J.. (1991). THE JOURNAL OF BIOLOGICAL CHEMISTRY Molecular Cloning of a Porcine Gene Syk That Encodes a 72-kDa Protein-Tyrosine Kinase Showing High Susceptibility to Proteolysis*. J. Biol. Chem. 266, 15790–15796. doi: 10.1016/S0021-9258(18)98478-4 1874735

[B55] Thoma G. Smith A. B. Van Eis M. J. Vangrevelinghe E. Blanz J. Aichholz R.. (2015a). Discovery and Profiling of a Selective and Efficacious Syk Inhibitor. J. Med. Chem. 58, 1950–1963. doi: 10.1021/JM5018863 25633741

[B56] Thoma,. G. Veenstra S. Strang R. Blanz J. Vangrevelinghe E. Berghausen J.. (2015b). Orally Bioavailable Syk Inhibitors With Activity in a Rat PK/PD Model. Bioorg. Med. Chem. Lett. 25, 4642–4647. doi: 10.1016/J.BMCL.2015.08.037 26320624

[B57] Turner M. Mee P. J. Costello P. S. Williams O. Price A. A. Duddy L. P.. (1995). Perinatal Lethality and Blocked B-Cell Development in Mice Lacking the Tyrosine Kinase Syk. Nature 378, 298–302. doi: 10.1038/378298A0 7477352

[B58] Van Der Spoel D. Lindahl E. Hess B. Groenhof G. Mark A. E. Berendsen H. J. C. (2005). GROMACS: Fast, Flexible, and Free. J. Comput. Chem. 26, 1701–1718. doi: 10.1002/JCC.20291 16211538

[B59] Veber D. F. Johnson S. R. Cheng H. Y. Smith B. R. Ward K. W. Kopple K. D. (2002). Molecular Properties That Influence the Oral Bioavailability of Drug Candidates. J. Med. Chem. 45, 2615–2623. doi: 10.1021/JM020017N/SUPPL_FILE/JM020017N_S.PDF 12036371

[B60] Wang X. Guo J. Ning Z. Wu X. (2018). Discovery of a Natural Syk Inhibitor From Chinese Medicine Through a Docking-Based Virtual Screening and Biological Assay Study. Molecules 23. doi: 10.3390/MOLECULES23123114 PMC6320911 30487406

[B61] Zoete V. Cuendet M. A. Grosdidier A. Michielin O. (2011). SwissParam: A Fast Force Field Generation Tool for Small Organic Molecules. J. Comput. Chem. 32, 2359–2368. doi: 10.1002/JCC.21816 21541964

