# Supplementary material for: 3D-QSAR-Based Pharmacophore Modeling, Virtual Screening, and Molecular Dynamics Simulations for the Identification of Spleen Tyrosine Kinase Inhibitors
Source: Front Cell Infect Microbiol. 2022 Jun 30;12:909111. doi: 10.3389/fcimb.2022.909111 (PMC9280624; doi:10.3389/fcimb.2022.909111)
Supplement: Supplementary file 1 [file DataSheet_1.pdf]

Supplementary Material

1 Supplementary Figures and Tables

1.1 Supplementary Figures

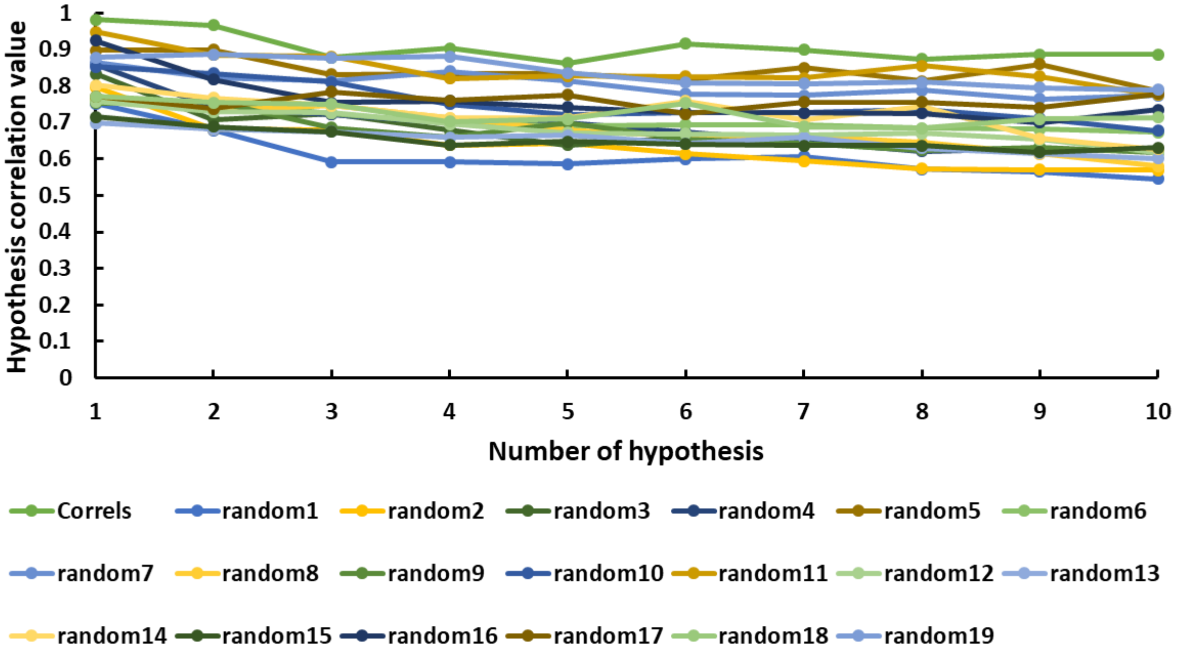

Supplementary Figure S1. Supplementary figure S1. The graphical representation of the difference between correlation values of initial spreadsheet (Correls) and 19 random spreadsheets.

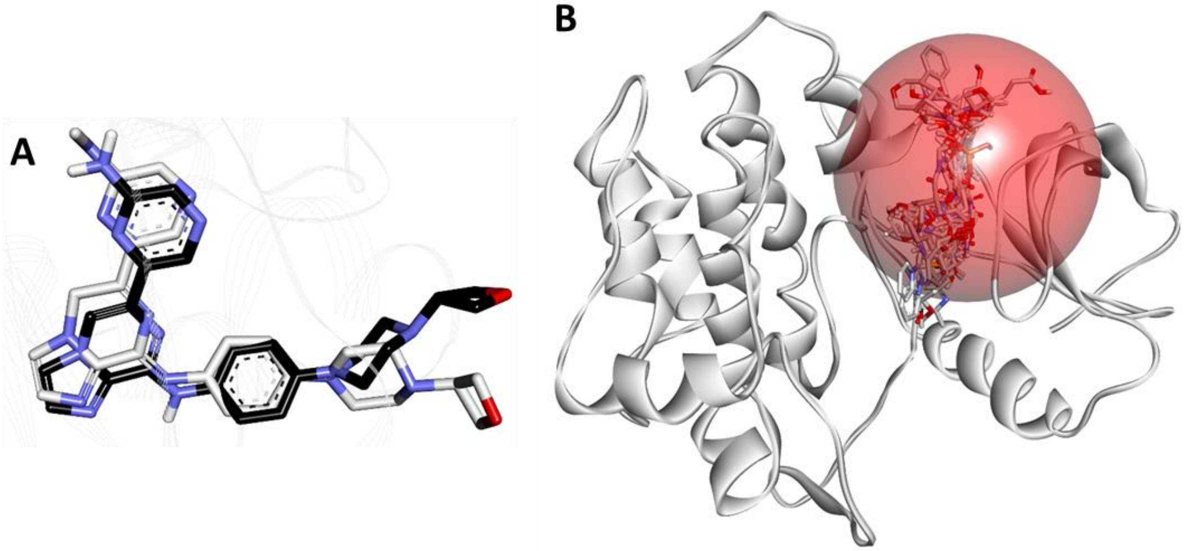

Supplementary Figure S2. Molecular docking analysis. A) Structure overlap between co-crystallized ligand (grey) and docked pose (black). B) The selected compounds from molecular docking inside the active site of SYK. The docking sphere was shown with red.

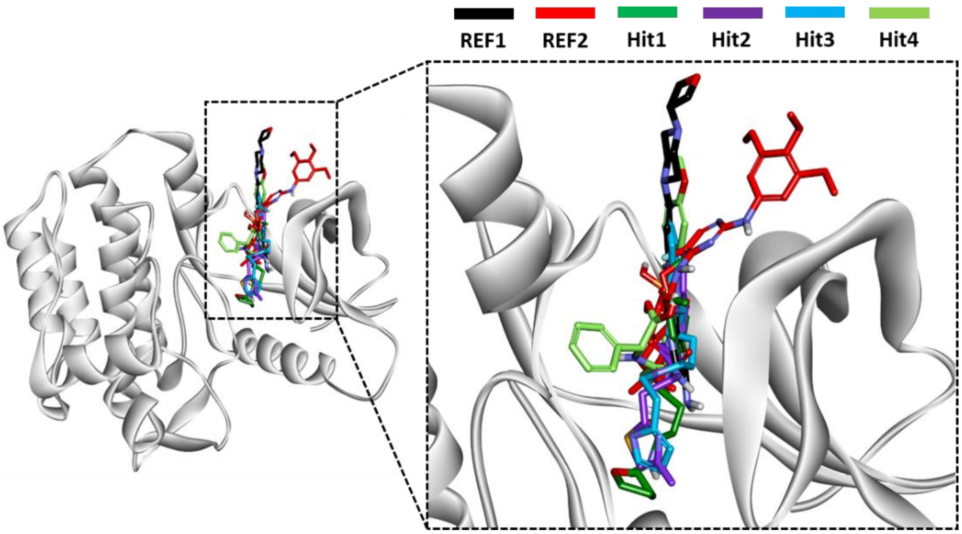

Supplementary Figure S3. The binding pattern of the hit compounds and REF inhibitors inside the active site of the SYK after MD simulations. The protein is shown with grey color solid ribbons.

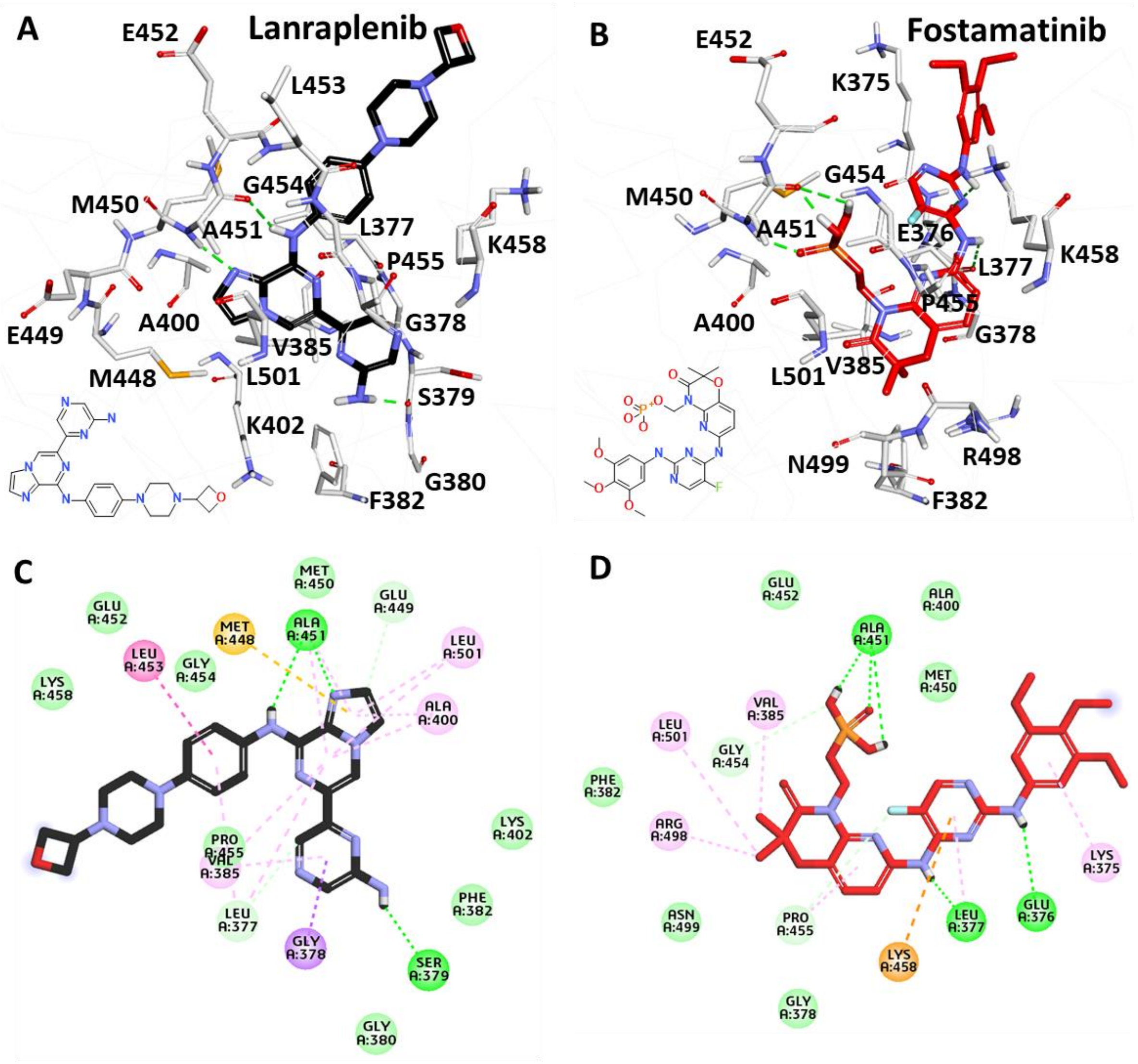

Supplementary Figure S4. The 3D and 2D binding modes of the REF inhibitors inside the active site of the SYK. In the upper half, protein in the background is shown with a grey color line representation. The protein residues involved in polar and non-polar interactions were shown in grey stick representations. The hydrogen bond, π-π, π-alkyl, π-cation, π-sulfur, and π-σ interactions are shown as green, pink, orange, yellow, and purple dash line, respectively.

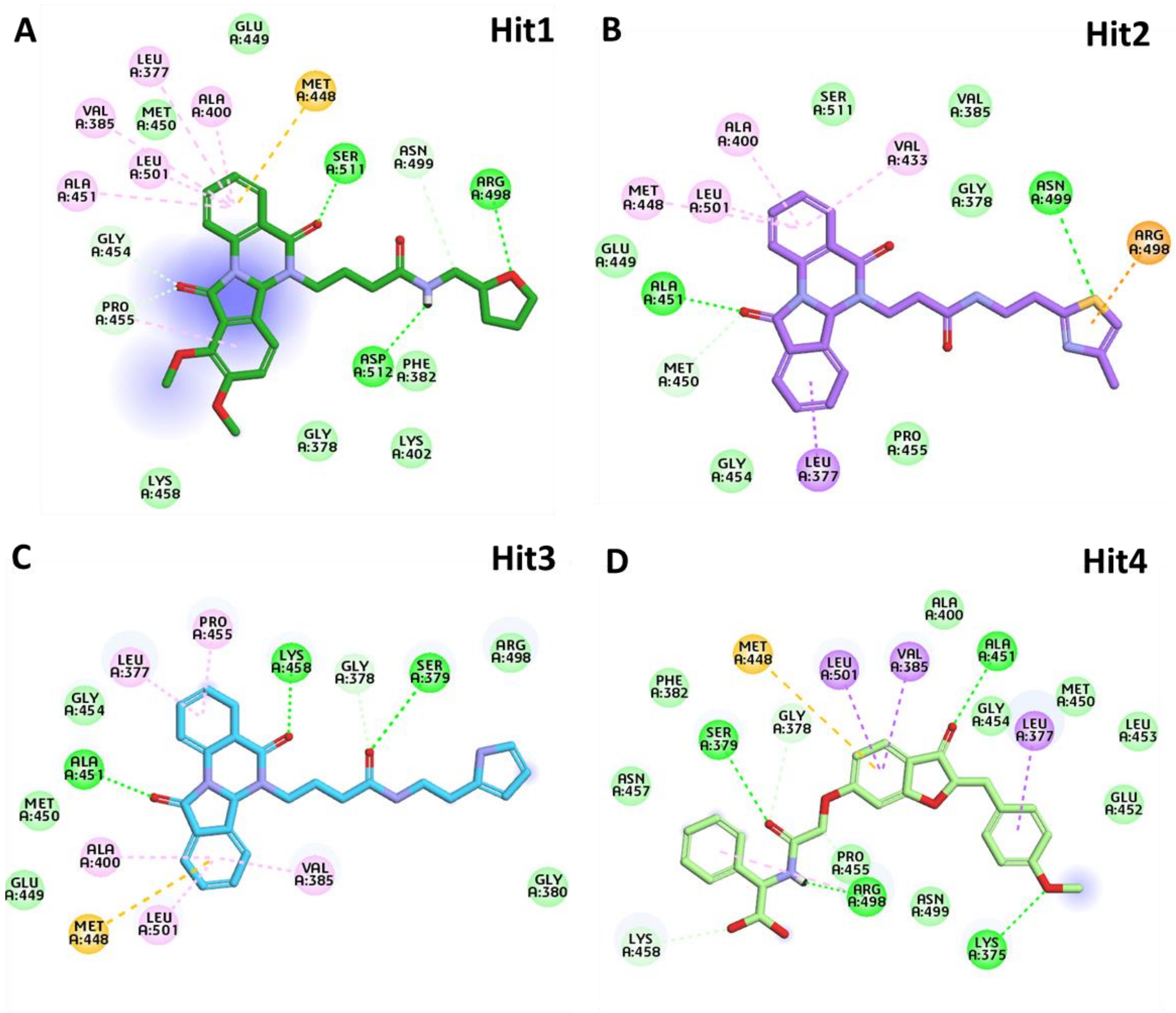

Supplementary Figure S5. The 2D binding mode of the selected hit compounds with SYK active site residues. Hit1 (A), Hit2 (B), Hit3 (C), and Hit4 (D) are shown with different color schemes in stick representation. The hydrogen bond, π-π, π-alkyl, π-cation, π-sulfur, and π-σ interactions are shown as green, pink, orange, yellow, and purple dash line, respectively.

1.2 Supplementary Tables

Supplementary Table S1. Chemical structures of test set compounds with their experimental activity (IC_{50}) values used for hypothesis validation.

Compound No.	IC_{50} (nmol/L)	Structure
1	5	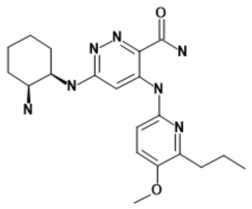
2	10	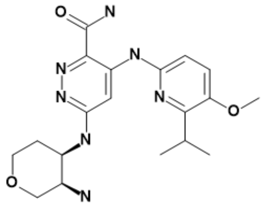
3	17	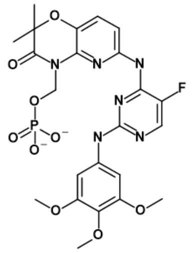
4	19	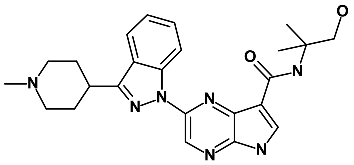
5	30	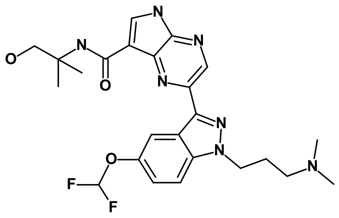

6	63	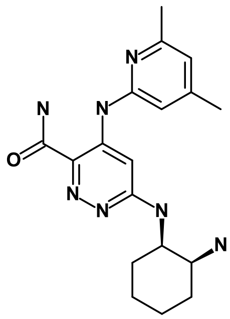
7	79	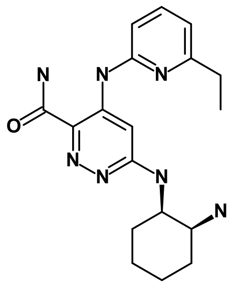
8	158	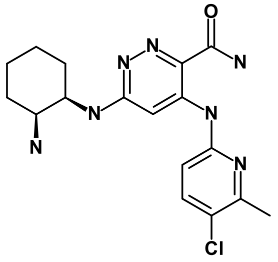
9	200	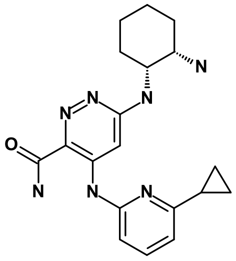
10	251	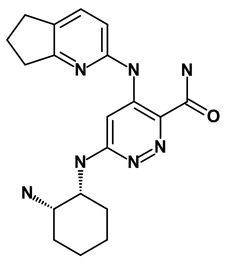

11	398	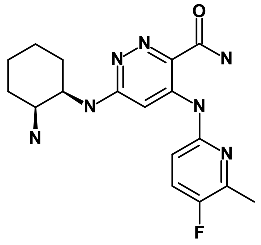
12	412	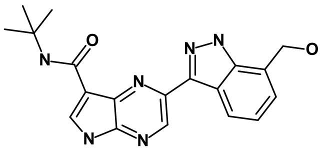
13	501	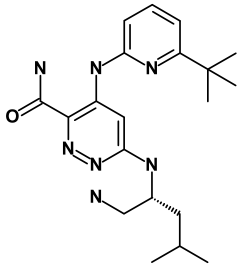
14	643	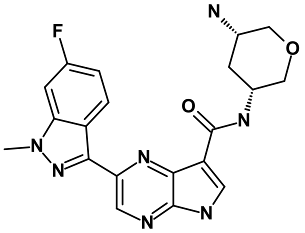
15	852	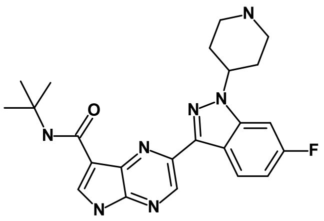
16	1430	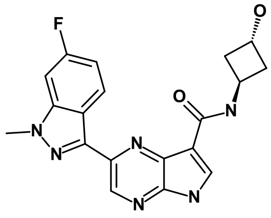

17	1770	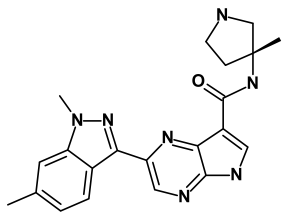
18	2580	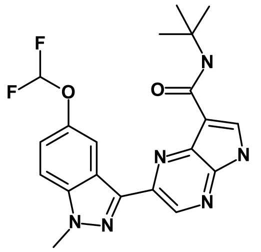
19	9950	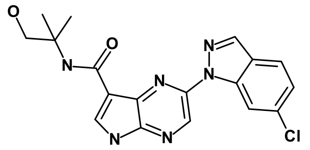

Supplementary Table S2. Detailed overview of experimental activities of test set compounds and their predicted activities based on Hypo1.

Compound No.	Fit Value	Experimental IC_{50} nmol/L	Predicted IC_{50} nmol/L	Error[a]	Experimental Scale[b]	Predicted Scale[b]
1	9.04	5	2.95	-0.59	+++	+++
2	9.19	10	2.05	-0.2	++	+++
3	8.89	17	4.16	-0.24	++	+++
4	8.22	19	19.31	+1.01	++	++
5	9.08	30	2.69	-0.08	++	+++
6	7.87	63	43.19	-0.68	++	++
7	7.71	79	62.12	-0.78	++	++
8	7.58	158	83.63	-0.52	++	++

9	7.93	200	37.74	-0.18	++	++
10	7.66	251	69.91	-0.27	++	++
11	7.69	398	66.04	-0.16	++	++
12	7.54	412	92.97	-0.22	++	++
13	8.07	501	27.32	-0.05	++	++
14	6.79	643	518.83	-0.8	++	++
15	7.84	852	46.57	-0.05	++	++
16	6.42	1,430	1216.30	-0.85	+	+
17	5.65	1,770	7122.39	+4.02	+	+
18	6.23	2,580	1868.10	-0.72	+	+
19	5.43	9,950	11924.90	+1.19	+	+

[a]Error, ratio of the (Predicted IC_{50} to the experimental IC_{50} or its negative inverse if the ratio is <1.

[b]Activity scale: IC_{50} <10 nmol/L = +++ (active), 10 nmol/L ≤ IC50 < 1000 nmol/L = ++ (moderate active), IC50 ≥ 1000 nmol/L = + (Inactive)

Supplementary Table S3. The molecular docking scores, hydrogen bond interactions with the SYK domain, and the binding free energies scores (MM-PBSA) of the selected compounds.

Database ID	GoldScore	ChemScore	H-bond Interactions	Binding free energy (kJ/mol)
ZINC98364146	77.50	-34.12	Ser379, Lys458, Asp512	-74.64+/-25.35
ZINC20590657	76.82	-32.81	Ala451, Lys458	85.84+/-20.62
ZINC19370937	76.71	-34.22	Ala451, Lys458, Asp512	95.79+/-16.17
ZINC98364453	76.19	-30.14	Asp512	-52.04+/-30.57
ZINC20590553	75.39	-34.43	Ala451, Lys458	84.91+/-19.52
ZINC98363696	74.81	-31.14	Asp512	-82.53+/-18.39
ZINC20589935	74.78	-34.61	Ala451, Lys458	77.13+/-20.24
ZINC98363910	74.73	-31.36	Asp512	-50.05+/-17.93
ZINC22788155	74.59	-31.70	Ala451, Lys458	75.42+/-21.26
ZINC98365358	74.44	-31.28	Ser379, Asp512	-105.30+/-13.45
ZINC32502133	73.89	-39.04	Ala451, Asp512	85.63+/-33.14
ZINC02123418	73.20	-22.92	Ala451, Asp512	-79.10+/-13.95
ZINC20533280	72.61	-33.40	Ala451, Lys458, Asp512	122.20+/-19.29
ZINC98365377	72.40	-25.37	Ala451, Asp512	-82.87+/-18.99
ZINC22930747	71.90	-32.96	Met448, Ala451, Lys458, Asp512	113.95+/-16.35
ZINC98364043	71.82	-26.83	Ala451, Asp512	-91.60+/-12.88
ZINC20759504	71.76	-25.77	Asp512	28.40+/-29.42
ZINC11865138	71.61	-33.35	Ala451, Asp512	130.07+/-22.67
ZINC20573473	71.50	-34.41	Ala451	78.42+/-14.98

ZINC04030012	71.32	-29.94	Lys375, Lys387, Lys402, Ala451, Glu452	-74.82+/-16.60
ZINC98363754	70.85	-29.63	Asp512	-86.13+/-17.20
ZINC22468262	70.72	-29.91	Als451	87.78+/-19.83
ZINC22468256	70.67	-31.61	Ala451, Lys458, Asp512	44.49+/-15.63
ZINC08789982	70.55	-30.79	Ala451, Asp512	-96.76+/-16.80
ZINC98364133	70.00	-29.55	Ala451, Lys458	-98.81+/-15.54
ZINC08790349	69.92	-30.33	Ala451, Gln462, Asp512	-86.56+/-20.66
ZINC98364834	69.30	-28.76	Asp512	-79.83+/-16.36
ZINC98363745	68.75	-30.74	Asp512	-111.11+/-20.13
ZINC09299943	68.67	-34.77	Ala451	70.87+/-12.84
ZINC12881584	68.50	-30.09	Lys402, Ala451, Asp512	-68.257+/-16.13
ZINC08789918	67.79	-28.94	Ala451, Asp512	-74.91+/-20.75
ZINC12885867	67.57	-33.36	Ala451, Asp512	-91.39+/-15.54
ZINC22785353	66.91	-32.04	Ala451, Asp512	90.12+/-23.42
Fostamatinib	66.62	-17.60	Ala451	-90.00+/-32.01
Lanraplenib	64.56	-28.99	Met450, Ala451, Asp512	-84.53+/-20.67

Supplementary Table S4. The chemical names of the identified scaffolds as SYK inhibitors.

Hits	IUPAC name
Hit1	4-[(6aS)-9,10-dimethoxy-5,11-dioxo-6aH-isoindolo[2,1-a]quinazolin-6-yl]-N-[[(2S)-tetrahydrofuran-2-yl]methyl]butanamide
Hit2	3-[(6aR,11S)-11-hydroxy-5-oxo-6a,11-dihydroisoindolo[2,1-a]quinazolin-6-yl]-N-[2-(4-methylthiazol-2-yl)ethyl]propanamide
Hit3	4-[(4aS,6aR)-5,11-dioxo-4a,6a-dihydro-4H-isoindolo[2,1-a]quinazolin-6-yl]-N-[2-(1H-pyrrol-2-yl)ethyl]butanamide
Hit4	(2R)-2-[[2-[(2S)-2-[(4-methoxyphenyl)methyl]-3-oxo-benzofuran-6-yl]oxyacetyl]amino]-2-phenyl-acetic acid
